# Repurposing the antimalarial pyronaridine tetraphosphate to protect against Ebola virus infection

**DOI:** 10.1371/journal.pntd.0007890

**Published:** 2019-11-21

**Authors:** Thomas R. Lane, Christopher Massey, Jason E. Comer, Manu Anantpadma, Joel S. Freundlich, Robert A. Davey, Peter B. Madrid, Sean Ekins

**Affiliations:** 1 Collaborations Pharmaceuticals, Inc., Raleigh, NC, United States of America; 2 Department of Microbiology and Immunology, University of Texas Medical Branch, Galveston, TX, United States of America; 3 Institutional Office of Regulated Nonclinical Studies, University of Texas Medical Branch, Galveston, TX, United States of America; 4 Sealy Center for Vaccine Development, University of Texas Medical Branch, Galveston, TX, United States of America; 5 Department of Virology and Immunology, Texas Biomedical Research Institute, San Antonio, TX, United States of America; 6 Departments of Pharmacology, Physiology, and Neuroscience & Medicine, Center for Emerging and Reemerging Pathogens, Rutgers University–New Jersey Medical School, NJ, United States of America; 7 SRI International, Menlo Park, CA, United States of America; Center for Disease Control and Prevention, UNITED STATES

## Abstract

Recent outbreaks of the Ebola virus (EBOV) have focused attention on the dire need for antivirals to treat these patients. We identified pyronaridine tetraphosphate as a potential candidate as it is an approved drug in the European Union which is currently used in combination with artesunate as a treatment for malaria (EC_50_ between 420 nM—1.14 μM against EBOV in HeLa cells). Range-finding studies in mice directed us to a single 75 mg/kg i.p. dose 1 hr after infection which resulted in 100% survival and statistically significantly reduced viremia at study day 3 from a lethal challenge with mouse-adapted EBOV (maEBOV). Further, an EBOV window study suggested we could dose pyronaridine 2 or 24 hrs post-exposure to result in similar efficacy. Analysis of cytokine and chemokine panels suggests that pyronaridine may act as an immunomodulator during an EBOV infection. Our studies with pyronaridine clearly demonstrate potential utility for its repurposing as an antiviral against EBOV and merits further study in larger animal models with the added benefit of already being used as a treatment against malaria.

## Author summary

To date there is no approved drug for Ebola Virus infection. Our approach has been to assess drugs that are already approved for other uses in various countries. Using computational models, we have previously identified three such drugs and demonstrated their activity against the Ebola virus *in vitro*. We now report on the *in vitro* absorption, metabolism, distribution, excretion and pharmacokinetic properties of one of these molecules, namely the antimalarial pyronaridine. We justify efficacy testing in the mouse model of ebola infection. We also demonstrate that a single dose of this drug is 100% effective against the virus. This study provides important preclinical evaluation of this already approved drug and justifies its selection for larger animal efficacy studies.

## Introduction

**Fig 1:**
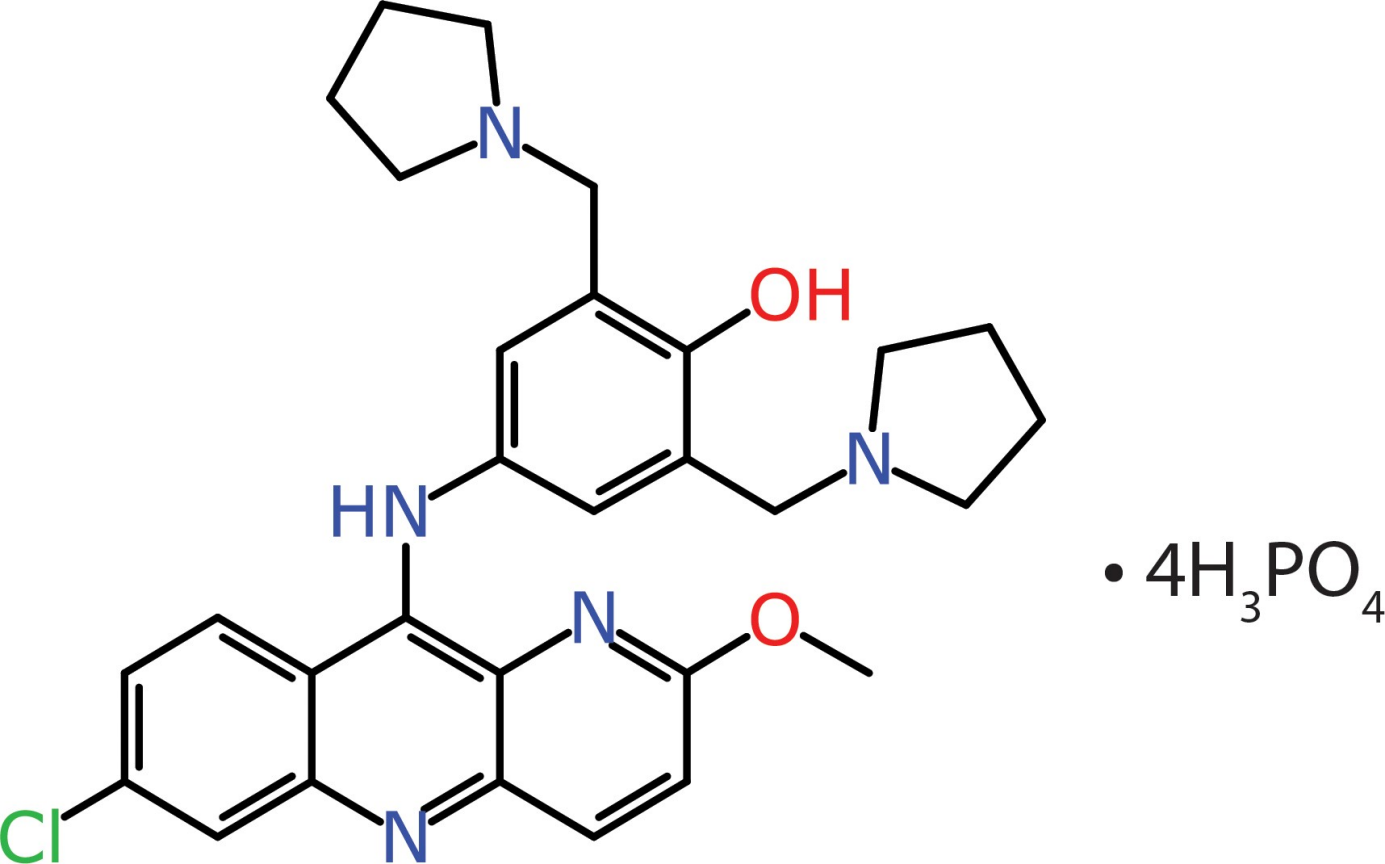
CPI-1058 (Pyronaridine tetraphosphate).

Ebola virus (EBOV) is a member of the family filovirus. Filoviruses are pathogenic against both humans and non-human primates and cause severe hemorrhagic fevers [1] with mortality rates as high as 90% [2, 3]. Recent outbreaks of EBOV in Africa have highlighted the need for new antiviral drugs for this and other emerging viruses [4]. The current ongoing outbreak in the Democratic Republic of the Congo, in which 2152 people have died at the time of writing (16th Oct 2019), again emphasizes this need for an antiviral treatment to compliment the ongoing vaccine and antibody-based approaches [5, 6]. There is a good understanding of the structure and function of the EBOV proteins [7] and while there is a broad array of therapeutics that have been investigated [8] there is still no current FDA approved drug for this disease. Recently, a clinical trial involving the investigation of multiple therapeutics against EBOV (NCT03719586) with ZMapp (a monoclonal antibody cocktail) [9]), remdesivir, MAb114 (a monoclonal antibody) [10]) and REGN-EB3 (monoclonal antibody combination) [11]) was described indicating REGN-EB3 and mAb114 had higher survival rates than ZMapp and remdesivir [12]. Current therapeutic discovery has focused on vaccines as they are likely to be the most promising approach [13]. However, the delivery and administration of temperature sensitive vaccines and antibody-based therapies to remote areas has challenges whereas a highly stable small molecule drug, that could be given orally would be ideal. Before the 2014 Ebola outbreak, several groups had performed high throughput screens and identified FDA approved drugs with *in vitro* growth inhibitory activities against EBOV [14, 15]. One of these, chloroquine was also tested in a mouse *in vivo* model and demonstrated a 90% survival rate (log rank p<0.001) [15]. This compound is however not ideal due to known toxicity [16, 17] and was inactive in guinea pig studies [18, 19]. There is hence considerable prior knowledge regarding small molecules that have activity against EBOV *in vitro* or in animal models [15, 20–25]. Compounds which are FDA-approved (or European EMA approved) drugs for other diseases [15, 24, 25], but additionally have activity against EBOV *in vitro* or *in vivo* may also represent useful starting points with the advantage that much is known regarding their absorption, distribution, metabolism and excretion (ADME) and toxicity properties. These repurposed drugs represent an advanced starting point for therapeutic development and approval compared with new chemical entities [26]. For example, high throughput screens have recently identified human topoisomerase II inhibitors [27] as VP35 inhibitors in EBOV. We have used Laplacian-corrected Naïve Bayesian classifier machine learning models based on data from a previously published high-throughput screen of 868 molecules using a viral pseudotype entry assay and an EBOV replication assay [15, 28] to perform a virtual screen of 2320 compounds and identified three active compounds [29]. Recombinant, infectious EBOV-encoding GFP was then used for testing the efficacy of these compounds using HeLa cells. One of these molecules is pyronaridine (EC_50_ range of 420 nM-1.14 μM in HeLa cells) [29, 30] (Fig 1), a component of the EU-approved antimalarial Pyramax, demonstrating the ability of a computational approach to identify compounds that are not widely known as antivirals as potential novel treatments against EBOV. While there is considerable data on pyronaridine as a chemotherapeutic for malaria [31], this study describes the first reported efficacy of pyronaridine to treat maEBOV-infected mice.

## Materials and methods

### Ethics statement

All work with maEBOV-challenged mice was approved by the University of Texas Medical Branch’s IACUC (IACUC protocol number 1805041 approved 5^th^ June 2018) and was done in accordance with all applicable sections of the Final Rules of the Animal Welfare Act regulations (9 CFR Parts 1, 2, and 3) and *Guide for the Care and Use of Laboratory Animals*: *Eighth Edition* (Institute of Laboratory Animal Resources, National Academies Press, 2011; the *Guide*). This work was conducted in UTMB’s AAALAC (Association for the Assessment and Accreditation of Laboratory Animal Care)-accredited GNL BSL4 laboratory.

### Chemicals and reagents

Pyronaridine tetraphosphate [4-[(7-Chloro-2-methoxybenzo[b][1,5]naphthyridin-10-yl)amino]-2,6-bis(1-pyrrolidinylmethyl)phenol phosphate (1:4)] [29] and tilorone dihydrochloride were purchased from BOC Sciences (Shirley NY).

### *In Vitro* ADME assays

*In vitro* ADME studies were performed by BioDuro (San Diego, CA) except for the CYP3A4 induction study which was performed by Eurofins Panlabs (Eurofins Panlabs, Inc. St. Charles MO).

### Bioanalytical Method for *In Vitro* ADME Studies

Test compounds were analyzed by reverse phase HPLC with a Kinetex 2.6μ C18 100A column (3.0 mm x 50 mm, Phenomenex (Torrance Ca)) using Shimadzu (Columbia, MD) LC-20AD system. The mobile phase consisted of solvent A (Water with 0.1% Formic) and solvent B (acetonitrile with 0.1% Formic). The MS detection was performed by using an API 4000 Q trap system. The amount of parent compound was determined on the basis of the peak area ratio (compound area to internal standard area).

### Kinetic solubility

396 μL of Universal Aqueous buffer (pH 7.4) was added to 4 μL of a 50 mM DMSO stock solution of pyronaridine. Wells were agitated for 4 h at 20°C and then filtered. The compound was then diluted to serial concentrations with DMSO, followed by serial dilutions with ACN: H2O (1:1) prior to LCMS analysis. The calculated concentration (μM) of soluble pyronaridine was determined in reference to a standard curve.

### Caco-2 permeability

Caco-2 cells (ATCC, Manassas, VA) were grown on 24-well (pore size: 0.4 μm) polycarbonate filters. The monolayers were pre-incubated with pre-warmed HBSS (Hank’s balanced salt solution) containing 2.5% HEPES buffer (pH 7.4) for 0.5 h at 37ºC. After pre-incubation, the buffer was removed and pyronaridine was added to reach a final concentration of 10 μM. 2% bovine serum albumin (BSA) was added to the receiver buffer for the study. The total volume was 400 μL for the apical (A) side and 1200 μL for the basolateral (B) side. For apical to basolateral transport study (A-B), 100 μL each was collected from both sides for sample analysis at the start of the assay and then 200 μL was collected from the apical side at 90 minutes (end of the study). The same timepoints and amounts were used for the basolateral to apical transport study (B-A).

The apparent permeability coefficient (Papp) was calculated from the following equation.

$$Papp(cm/\sec)=\frac{v•\partial C/\partial t}{A•C}$$

Where:

v = Volume of the receiver cell

A = Exposed surface area (0.64 cm^2^)

C = Initial donor concentration

∂*C*/∂*t* = Change in receiver concentration over time.

### Human CYP inhibition

Human liver microsome solution (0.2 mg/ml) (Sekisui Xenotech, Kansas City, KS), along with substrate, was aliquoted into a 0.05 M phosphate buffer (pH = 7.4) in 1.1 ml tubes. Study samples (containing either control inhibitor or test compound) were into added into the tubes, vortexed gently and pre-incubated for 5 min at 37°C. 20 μL of NADPH solution was aliquoted into all tubes, then vortexed to start the reaction and to assure adequate mixture of the NADPH. After mixing, the tubes were incubated for 20 min at 37°C in a shaking water bath and then quenched in 300 ml formic acid/acetonitrile solution. After quenching the samples were vortexed vigorously for 1 min and centrifuged at 4,000 rpm for 15 min (4°C). 100 μL of supernatant was transferred to 0.65 ml tubes, for LCMS analysis by the bioanalytical method described earlier. The CYP450 substrates and control inhibitors for each enzyme was as follows: CYP1A2 (phenacetin, naphthoflavone), CYP2C9 (diclofenac, sulfaphenazole), CYP2C19 (omeprazole, tranylcypromine), CYP2D6 (dextromethorphan, quinidine), CYP3A4 (midazolam, ketoconazole).

### Mouse liver microsome stability

Mouse liver microsome solution (197.5 μL, 1 mg/ml protein concentration) (BioIVT, Westbury, NY) was aliquoted into 1.1 ml tubes, to which 2.5 μL of positive control and pyronaridine stock solutions (100 μM in DMSO) were added. The tubes were vortexed gently, pre-incubated for 5 min at 37°C, then 50 μL of 5 mM NADPH or LM buffer (no NADPH buffer) was added into the tubes. For analysis, an aliquot of 15 μL was removed from each tube at 0, 5, 15, 30 and 60 min (without-NADPH reaction:0, 30 and 60 min) and quenched with 300 μL of 25 ng/ml propranolol in acetonitrile. Samples were vigorously vortexed for 1 min and then centrifuged at 4,000 rpm for 15 min at 4°C. 100 μL of supernatant from each sample was transferred to 0.65 ml tubes for LCMS analysis. The amount of parent compound was determined on the basis of the peak area ratio (compound area to IS area) for each time point. Clearance rates were calculated by the equation:

CL_int_ (μL/min/mg protein) = Ln (2)*1000 /T_1/2_ / Protein Conc.

### Protein binding in plasma

The donor side of dialysis inserts were filled with 200 μL plasma (human (Cat#HUMANPLK21804231) and mouse (MSE00PLK2YNN) plasma from BioIVT, (Westbury, NY)) containing 5 μM pyronaridine and 0.5% of DMSO and the receiver side of the dialysis inserts was filled with 350 μL of PBS buffer (100 mM, pH 7.4). The prepared dialysis apparatus was placed in a shaker (37°C, 100 rpm) for 5 hours. Two tubes with plasma containing 5 μM pyronaridine were also prepared for stability test, one tube was placed in the freezer (4°C) for 5 h, and the other tube was placed in shaker (3°C, 100 rpm) for 5 h. Samples were collected from the donor and receiver sides of each dialysis insert. The same volume of blank plasma was added to buffer samples and blank buffer to plasma samples to make sure all sample mixtures contain 50% plasma and 50% buffer. 50 μL of each sample was mixed with 300 μL of acetonitrile containing 25 ng/ml internal standard (propranolol). All samples were vortexed for 1 minute and then centrifuged at 4000 rpm, 4°C for 15 min. 100 μL of the supernatant was transferred to 0.65 ml tube for LCMS analysis. The amount of compound was determined on the basis of the peak area ratio (compound area to internal standard area) for the two sides, and protein binding is determined using the following equations: %Bound = 100 x([Area Ratio of Donor] 5h *5 - [Area Ratio of Receiver] 5h) / ([Area Ratio of Donor] 5h *5). The percentage remaining at 37°C after 5h was calculated on the basis of the amount measured at 0°C after 5h.

### *In Vitro* metabolite identification of Pyronaridine in Mouse liver microsomes

The total volume for incubation was 1000 μL. A 1000 μM DMSO solution of pyronaridine was spiked into 50 mM KH_2_PO_4_ (pH 7.4) buffer containing liver microsome (BioIVT, Westbury, NY) at a concentration of 1 mg/mL. The reaction was initiated by the addition of 5 mM NADPH to the reaction mixture. The final concentration of pyronaridine was 10 μM. After 0min, 30min, 60min and 120 min incubation at 37ºC, an aliquot of 200 μL was removed and 600 μL of acetonitrile was added to quench the reaction. The resulting mixture was centrifuged at 4,000 rpm for 15 min. The resultant supernatant was dried at N_2_ stream, the resultant residue were reconstituted with 300 μL 30% acetonitrile/H_2_O (v/v) before LC-MS/MS analysis. The supernatant was used for LC-MS/MS analysis. All separations were performed on a Kinetex 2.6u C18 100A column (3.0 mm * 50 mm) at room temperature with a flow rate of 0.6 mL/min. Mobile phase A consisted of 0.1% formic acid in water and mobile phase B consisted of 0.1% formic acid in acetonitrile. Chromatography used a step gradient by maintaining 2% mobile phase B for 3 minute, 2 to 20% mobile phase B over 15.0 minute, 20 to 50% mobile phase B over 17 minute, 50 to 90% mobile phase B over 3 minutes, remaining 90% mobile phase B for 5 minute, then re-equilibration back to 2% B at 25 min. The total run time was 30 minutes. For all samples, 5 μL aliquot of sample was injected. The mass spectrometer (API-4000 QTrap, Applied Biosystems/MDS SCIEX Instruments, Foster City, CA) was operated in positive ion multiple reaction monitoring mode (MRM).

### CYP3A4 induction

As CYP3A4 induction is a key issue for large hydrophobic molecules pyronaridine was tested in cryopreserved hepatocytes (BioIVT, Westbury, NY) (at 1, 10, 100μM) using 10μM rifampicin as positive control and midazolam as the substrate for the enzyme (Eurofins Panlabs, Inc. St. Charles MO).

### *In vitro* data for EBOV and combination testing

Ebola virus (Mayinga) was used for testing efficacy of compounds. All viral infections were done in the BSL-4 lab at Texas Biomedical Research Institute. Briefly, 4,000 HeLa cells (Ambion, Austin, TX) per well were grown overnight in 384-well tissue culture plates, the volume of culture medium was 25 μL. On the day of assay, test compounds were diluted to 8 times the final desired concentration in complete medium on separate plates. The compounds from different plates were mixed into a new plate to achieve 4 times the desired concentration. Compound mixture was then added to in equal volume to medium overlaying the cells, thereby achieving 2 times the desired concentration. Treated cells were then incubated at 37°C in a humidified CO2 incubator for 1 h. Final concentrations of pyronaridine 25.00, 12.50, 6.25, 3.13, 1.56, 0.78, 0.39, 0.20, 0.10, 0.05, 0.02 and 0.01 μM were achieved in rows A-L upon addition of 25 μL of infection mix containing Ebola virus. The same concentrations of Tilorone were achieved in columns 1–12. The final concentrations can be represented in the grid (S2 Table). Infections were done to achieve a MOI of 0.01 to 0.25. The virus challenged cells were incubated for 24 h. 24 h post infection cells were fixed and inactivated by immersing the plates in formalin for 24 h at 4°C. Plates were washed 3x with PBS. EBOV infected cells were stained for viral antigen using previously described protocols [32]. Nuclei (blue) and infected cells (green) were counted using CellProfiler software. Total number of nuclei (blue) was used as a proxy for cell numbers and a loss of cell number was assumed to reflect cytotoxicity.

The BRAID analysis [33] service calculates synergy by fitting data to a seven-variable function. The variable κ represents a quantitative synergy value where κ < 0 implies antagonism, κ  = 0 implies additivity, and κ > 0 implies synergy. To assess if the combined inhibitory effect of tilorone and pyronaridine on EBOV was synergistic, additive, or antagonistic we performed a checkboard assay with pyronaridine and tilorone at various combined concentrations (Fixed pyronaridine/tilorone concentrations of 0.012, 0.024, 0.049, 0.098, 0.195, 0.391, 0.781, 1.562, 3.125, 6.25, 12.5, or 25 μM) in HeLa cells.

### Virus screening

Pyronaridine tetraphosphate was also tested (using the NIAID DMID services) against representatives of the herpesviridae, bunyaviridae, togaviridae, arenaviridae, flavivirdae, picornaviridae, poxviridae, hepatic viruses, respiratory viruses and other viruses.

### Test Article Preparation for *In Vivo* Studies

Dose formulations for pyronaridine and tilorone were prepared under yellow light by mixing the appropriate amount of test article in melted Kolliphor HS 15 (Solutol) (20% final volume) using a vortex mixer for 30 s. The remaining sterile water (Gibco) was added, and the formulations were mixed using a vortex mixer for 30 sec– 5 min until the compound was visually dissolved and then sonication for 25 min. The final 20% Kolliphor HS 15 dose formulations were observed to be clear, reddish solutions. The favipiravir dose formulations for oral and interperitoneal administration were 0.5% CMC and 74.6 mg/ml meglumine (pH 8.5), respectively. The final solution was a clear, pale yellow.

### *In Vivo* pharmacokinetics

Pyronaridine was administered to 7- to 8-week-old male and female BALB/c mice (Charles River) by i.p. injection for the dose range finding study. Animals were observed immediately post-dose and twice daily up to 72 h. Clinical observations were performed immediately post-dose and twice daily up to 72 h post-dose. For PK studies, blood from saphenous vein puncture of 8- to 9-week-old BALB/c male mice (Shanghai Lingchang Biotechnology Co., Ltd.) was collected in tubes containing K_2_EDTA, processed to plasma, and stored frozen at -20°C before 168 h and -80°C after 168 hr or analyzed in a short time after the sample was taken. The drug level of pyronaridine was determined in collected plasma samples using the bioanalytical method described below. The plasma drug level data were analyzed using Phoenix WinNonlin (Version 8.0) to perform non-compartmental analysis. Parameters determined were time to maximum concentration of drug in serum (t_max_), C_max_, t_1/2_, AUC_inf_ and AUC_last_. The time points used to calculate t_1/2_ were 336, 504, 672 hr. The software also calculated the standard deviations (SD) for t_1/2_, t_max,_ AUC_inf_ and AUC_last_. The standard error mean (SEM) was calculated from this by dividing the SD by the square root of the sample size.

### Bioanalytical method

The extraction method for analysis and quantitation of pyronaridine in mouse plasma was 0.2 ml of an internal standard solution (5.0 ng/ml terfenadine in acetonitrile/MeOH (1:1, v/v) added to 0.01 ml mouse plasma in a 1.1-ml microcentrifuge tube. 0.02 ml of DMSO was added to all samples then vortexed for 1 min and then centrifuged at 4000 rpm for 15 min. The supernatant was then collected and diluted 10x with MeOH:water (1:1) for injection. For the analysis of samples, chromatographic separation of pyronaridine and IS from endogenous interferences was performed on a Waters XSelect C18 5 μm column (2.1 mm * 50 mm) at room temperature with a flow rate of 0.7 mL/min. Mobile phase A consisted of 5mM NH4OAC (0.1% FA) and mobile phase B consisted of 0.1% formic acid in acetonitrile. Chromatography used a linear gradient by maintaining 2.0% mobile phase B for 0.4 minute, 2 to 95% mobile phase B over 1.6 minute, followed by a 95% mobile phase B wash for 0.2 minute and a re-equilibration at 2% B for 0.79 minute. Quantitation was performed using MS/multiple reaction monitoring (MRM) analysis on an AB Sciex API 5500 Q-Trap mass spectrometer (Sciex, Framingham, MA). Pyronaridine was measured by m/z 518.36/447.30 Da and terfenadine by m/z 472.40/436.40 Da. The LC-MS/MS data were acquired, peak areas were integrated, the calibration line regression was calculated, and the final concentrations were generated using AB Sciex Analyst software.

### Virus strains

For *in vivo* experiments, a well-characterized mouse-adapted Ebola virus stock (Ebola virus *M*. *musculus*/COD/1976/Mayinga-CDC-808012); first reported by Bray et al. [34] was used for all efficacy studies. All work involving infectious maEBOV was performed at the Galveston National Laboratory (GNL) biosafety level (BSL) 4 laboratory, registered with the Centers for Disease Control and Prevention Select Agent Program for the possession and use of biological select agents.

### *In Vivo* efficacy clinical observations and scoring

Animals were monitored daily by visual examination. Clinical scoring and health assessments were performed and documented at each observation using a quantitative assessment of pain and distress scoring system. Animals are scored based on the following observations: 1 –healthy; 2 –lethargic, ruffled fur; 3 –ruffled fur, lethargic, hunched posture, orbital tightening; 4 –ruffled fur, lethargic, hunched posture, orbital tightening, reluctance to move when stimulated, paralysis OR ≥20% weight.

Once animals reached a clinical score of 2, they were observed twice daily with 6–8 hours between observations. Animals in advanced disease (score of 3) were observed a third time. The third observation occurred 4–6 hours after the afternoon observation. Animals that scored a 4 were humanely euthanized. All surviving animals were humanely euthanized on Study Day 21. Mice were weighed daily through Study Day 7. Following this period, and for the remainder of the study, animals were weighed every 3 days and monitored at least once per day for the development of clinical signs.

### Virus administration

Virus (100 PFU in 100 μL) was administration via ip injection. The challenge suspension was back titered to confirm the dose. For the efficacy study the first treatment for all groups occurred via i.p. injection using 100 μL of the test article given 1 h ± 15 min post challenge. The variation in treatment for the maEBOV window study was that the first dosing of the test article was given 2 h ± 15 min, 24 h ± 60 min, or 48 h ± 60 min post-challenge.

### Viral load determination

Six mice (3 male and 3 female) form each group were euthanized on day 3 and serum harvested. When possible, serum was also harvested from mice that met the euthanasia criteria. Serum harvested for plaque assay analysis was stored frozen (in an ultralow [i.e., -80°C] freezer) until the conclusion of the in-life portion of the animal study after which samples were batch processed. Collected sera for qRT-PCR analysis were added to TRIzol LS Reagent then stored frozen (in an ultralow freezer) until the conclusion of the in-life portion of the animal study after which samples were batch processed in the plaque and qRT-PCR assays.

### EBOV plaque assay

Serum samples were removed from frozen storage, thawed, and serially diluted in filtered-sterilized dilution medium (MEM/1% heat-inactivated fetal bovine serum/1% Penicillin-Streptomycin (PS)) for analysis. Samples were titered on Vero CCL-81 cells in 12-well plates by standard protocols. Briefly, log dilutions of serum samples were added to 80% confluent monolayers and rocked every 15 min. At 1 h post-infection cells were overlaid with semi-solid overlay of MEM, 0.5% methylcellulose, 2% HI-FBS, and 1% PS. Ten days post-infection the overlay was removed and monolayers were stained and fixed using 10% neutral buffered formalin with crystal violet. Plaques were enumerated and virus titers as plaque forming units (PFU) per ml calculated. For this assay, the limit of detection in this assay was 50 PFU/mL. For statistical analysis and graphing all values less than the LOD were assign a value of one half the LOD.

### EBOV qRT-PCR assay

On the day of collection, harvested serum (target of 0.05 ml) was added to TRIzol LS Reagent (5X volume; i.e., 0.25 ml) and mixed thoroughly. This solution was stored frozen as already described. For processing, samples were removed from frozen storage, thawed, and processed for RNA extraction and purification using the Zymo Direct-zolTM RNA Mini Prep kit. RNA samples were analyzed via quantitative RT-PCR using QIAGEN QuantiFast Probe RT-PCR kit, Forward primer: 5’- TTT TCA ATC CTC AAC CgT AAg gC-3’, Reverse primer: 5’- Cag TCC ggT CCC AgA ATg Tg-3’ and Probe: 5’-6FAM- CAT gTg CCg CCC CAT CgC TgC-MGBNFQ-3’. All reactions were performed on a Bio-Rad CFX96TM Real-Time PCR Detection System. For quantification purposes, a HPLC-purified synthetic EBOV RNA standard derived from the conserved EBOV glycoprotein (GP) gene was used. For this assay, the lower limit of quantification (LLOQ) was defined as 1.00E+03 GEq/μL because this was the lowest point tested on the standard curve. The upper limit of quantification was defined as 1.00E+10 GEq/μL as this was the highest tested point in the standard curve. For statistical analysis and illustration, samples below the LLOQ were assigned a value of one half the LLOQ.

### Cytokine panels

The Bio-Plex Pro Mouse Cytokine 23-plex panel was purchased from Bio-Rad Laboratories, Inc. (Hercules, California). This panel allowed for the serum quantification of the following molecules: Eotaxin, G-CSF, GM-CSF, IFN-γ, IL-1α, IL-1β, IL-2, IL-3, IL-4, IL-5, IL-6, IL-9, IL-10, IL-12 (p40), IL-12 (p70), IL-13, IL-17A, KC, MCP-1, MIP-1a, MIP-1b, RANTES and TNF-α. Quantification of IFN-α and IFN-β used the 2-plex mouse ProcartaPlex Panel (ThermoFisher Scientific, Waltham, MA). The protocols used were as per the manufacturer’s recommendations. All cytokines were quantified by the Bio-Rad Bio-Plex 200.

## Results

### Pyronaridine antiviral activities *In vitro*

Pyronaridine was evaluated *in vitro* for its anti-EBOV activity (Zaire strain) in the type I IFN-deficient Vero 76 cell line [35, 36] and showed a lack of antiviral activity at any concentration below the 50% cytotoxicity concentration (CC_50_ = 1.3 μM, S1 Table). This is in contrast to prior data in HeLa cells showing selectivity (EC_50_ = 0.42–1.12 μM, CC_50_ = 3.1 μM) [29, 30]. These observations support the hypothesis that pyronaridine’s antiviral activity are likely acting through or on the type I IFN-related innate immunity pathway in a similar manner to tilorone, which responds similarly in these IFN-deficient cells [37]. We also tested a combination of pyronaridine with tilorone in HeLa cells and evaluated the data with the BRAID model by Shelat and colleagues [33] (S2 Table). A calculated κ  = 0.488 (95% CI = -0.543 to 8.18) indicated that the EBOV inhibitory effects of these compounds are likely synergistic with each other in HeLa cells (S1 Fig), although the large 95% CI suggested that the confidence of this interpretation is limited. Analysis was performed following the removal of data with cytotoxic experimental conditions (data where the post-treatment cell count was reduced below 50% of the average control) and after excluding these data there are limited datapoints, making an accurate EC_50_ extrapolation of pyronaridine difficult. The BRAID analysis shows potentiation of the EC_50_ of tilorone by pyronaridine, but due to toxicity of pyronaridine in the checkboard assay the EC_50_, and therefore the potentiation of pyronaridine, could not be accurately extrapolated. Independently, pyronaridine also demonstrated no apparent selectivity (SI_50_ > 10) for selected additional viruses (the majority were tested in Vero 76 cells) except for the human norovirus (GT1 strain) which was tested in HG223 cells. Pyronaridine appeared selective for human norovirus (EC_50_ = 3.5 μM and CC_50_ >100 μM, S1 Table) and in a similar activity range as the positive control 2’C-methyl cytidine (6.5 μM and CC_50_ > 300 μM). It is noted that this was the only viral assay that was tested using HG223 cells, which may suggest that this may be a cell-specific effect.

### Pyronaridine *in Vitro* ADME properties

*In vitro* absorption, distribution, metabolism, excretion (ADME), are important molecule properties that are assessed early in the drug development process [38, 39] to identify any potential liabilities and pharmacokinetic problems which could limit *in vivo* use [40, 41]. Despite Pyramax being an approved drug combination (with artesunate), there is little ADME data for pyronaridine in the mouse that has been published in peer reviewed journals. We therefore evaluated the kinetic solubility [42], human CYP inhibition [43], metabolic stability [44], Caco-2 permeability [45], and plasma protein binding [46] of pyronaridine prior to generating pharmacokinetics data (Table 1). The inhibition of CYP2D6 appeared to be the only Cytochrome P450 interaction of this compound. CYP3A4 induction in cryopreserved human hepatocytes suggested that at 10μM induction was minimal (1.4x) compared to rifampicin (6.6x) as a control. These *in vitro* ADME characteristics suggest that pyronaridine is predicted to be soluble, moderately stable, has good absorption based on Caco-2 data, will not be actively pumped out of cells by the efflux transporter P-glycoprotein (P-gp) and is unlikely to be a potent CYP3A4 inducer. After 120 min of incubation of pyronaridine tetraphosphate with mouse liver microsomes, five metabolites were identified (S3 Table, S2–S4 Figs) which primarily represent oxidation or reduction of the parent compound. The major metabolite was oxidation of one of the pyrrolidine rings. These *in vitro* ADME characteristics supported further investigation of pyronaridine in pharmacokinetics studies.

**Table 1:** ADME properties for Pyronaridine tetraphosphate.

Pyronaridine	
ADME property	Data
**Solubility**	168 μM at pH 7.4
**CYP inhibition**	1A2, 2C9, 2C19 (>50 μM), 3A4 (42.9 μM), 2D6 (2.23 μM)
**Mouse liver** **microsomes**	t_1/2_ = 173 min, CL_int_ = 4 μL/min/mg protein
**Guinea Pig liver microsomes**	t_1/2_ = 44.4 min, 15.6 μL /min/mg protein
**Non-Human primate liver microsomes**	t_1/2_ = 140.1 min, 4.9 μL /min/mg protein
**Human liver microsomes**	T_1/2_ = 169.7 min, 4.1 μL /min/mg protein
**Mouse plasma protein binding**	96.5%
**Human plasma protein binding**	95.1%
**Caco-2**	Papp A-B = 6.46; B-A = 4.8 (x10^-6^ cm/s) Efflux ratio = 0.74
**CYP3A4 Induction**	1.5x at 10 μM

### Mouse dose range-finding toxicity

To assess the tolerance of pyronaridine and to select dose groups for pharmacokinetics studies, the drug was given to male and female BALB/c mice as a single dose by intraperitoneal (i.p.) administration (S4 Table). The compound was formulated in 20% Kolliphor HS 15 (Solutol) in sterile water. Clinical observations were initiated immediately post-dose and twice daily up to 72 hrs post-dose. In the pyronaridine dose groups, dehydration, hunched posture and ruffled fur were observed at 50 and 100 mg/kg. At the higher dose level of 300 mg/kg, ataxia, dehydration, hunched posture, ruffled fur and hypoactivity preceded a moribund euthanasia for 2 male and 2 female mice. In addition, 1 male and 1 female were also found deceased in the 300 mg/kg group on Day 1. Based on these results, the maximum tolerated dose (MTD) for a single pyronaridine dose was determined as 100 mg/kg, with the noted adverse effects at lower doses.

### Mouse pharmacokinetics evaluation of pyronaridine

**Fig 2:**
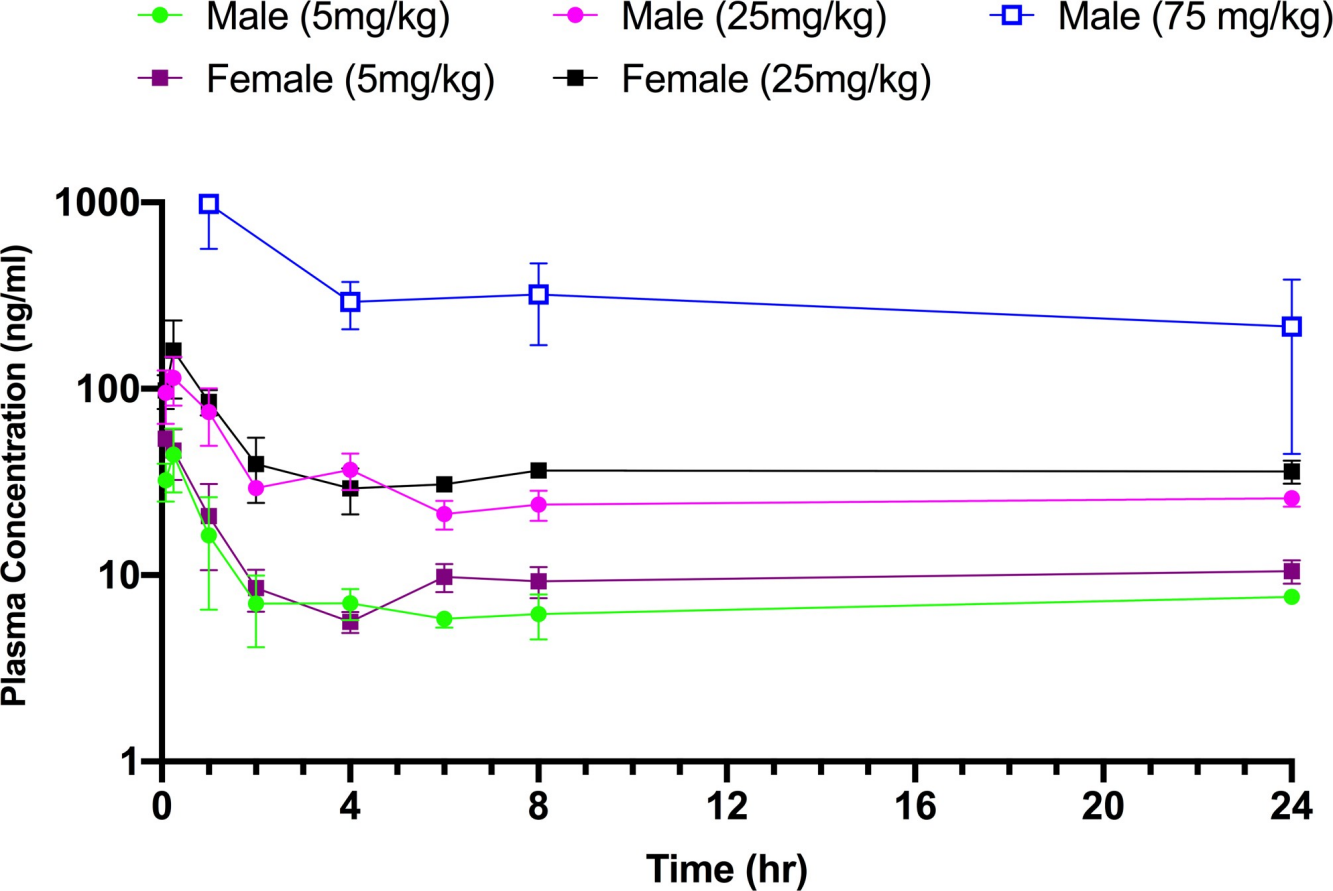
Pharmacokinetics of i.p. dosed pyronaridine in mice. The mean plasma concentration (± SD) vs. time profile from a single dose shows a dose-dependence plasma concentration.

**Fig 3:**
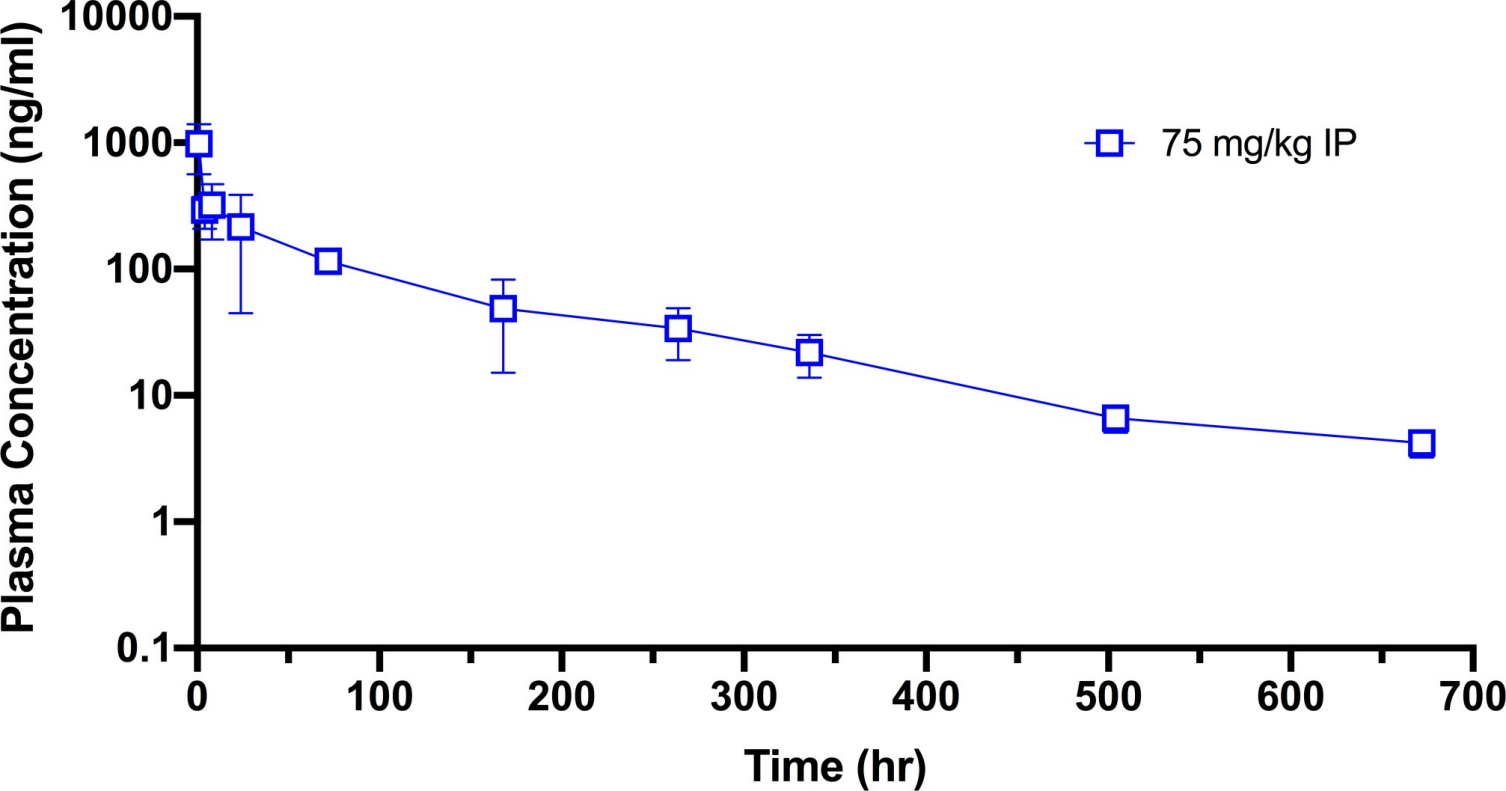
Mouse pharmacokinetics for i.p. dosed pyronaridine (75mg/kg): The mean plasma concentration (± SD) vs. time profile from a single dose.

Guided by the dose range-finding study, the pharmacokinetics of pyronaridine in mice were initially assessed at 5 and 25 mg/kg (n = 24; 12 male, 12 female), an amount well below the MTD. Each dose was administered by i.p. injection with the same vehicle (20% Kolliphor HS 15). Blood was collected from the treated mice at 5 and 15 min and at 1, 2, 4, 6, 8, and 24 h post-dose for processing of plasma. All samples were analyzed, and drug levels were measured by liquid chromatography-tandem mass spectrometry (LC-MS/MS) with a lower limit of quantitation (LLOQ) of 5.0 ng/mL. In the pyronaridine dose groups, ruffled fur and eye squinting were observed at 5 mg/kg and 25 mg/kg. After an initial rapid absorption phase, the pyronaridine plasma profile exhibited a distribution phase to about 2 hrs, then a prolonged phase with plasma drug concentrations remaining essentially unchanged, or slightly higher (Fig 2). All samples contained measurable levels of pyronaridine though 24 hrs (LLOQ = 5 ng/ml). Plasma concentrations of pyronaridine in male and female mice were not significantly different (p>0.05). In these initial sets of experiments the terminal phase was not sufficiently linear to accurately calculate the half-life (t_1/2_) and other elimination phase parameters. The time course was therefore elongated to accommodate the long t_1/2_ of pyronaridine (Fig 3). After an initial i.p. administration of 75 mg/kg of pyronaridine, blood was collected from the treated mice at 1, 4, 8, 24, 72, 168, 264, 336, 504, 672 h post-dose for processing to plasma (LLOQ = 1 ng/ml). The plasma drug levels were analyzed using noncompartmental modeling allowing for the calculation of pharmacokinetic parameters (Table 2). Pyronaridine plasma levels reached the peak between 0.25 and 1 hr. The elimination-phase t_1/2_ was calculated as 146 hr, comparable to that in humans of 195–251 h [47, 48]. Exposure to pyronaridine, based on maximum concentration of unbound drug in plasma (C_max_), area under the concentration-time curve from time zero to the last measurable concentration (AUC_last_), and area under the concentration-time curve from time zero to infinity (AUC_inf_), increased with the dose level in both male and female mice (at 5 mg/kg and 25 mg/kg doses). CL/F (clearance) and V/F (volume of distribution) were not corrected for absolute bioavailability (F) (Table 2).

**Table 2:** Pharmacokinetics data in mice treated with pyronaridine. NC = not calculated.

					C_max_ (ng/ml)		AUC_last_ (h*ng/ml)		
Dose (mg/kg)	Sex	T_1/2_ (h)	SE	T_max_ (h)	Mean	SE	Mean	SE	AUC_inf_ (h*ng/ml)
5	M	NC		0.25	44.4	9.58	192	12.2	NC
5	F	NC		0.083	54	2.44	257	13.5	NC
25	M	NC		0.25	115	19.3	713	34.2	NC
25	F	NC		0.25	160	41.4	956	45.5	NC
75	M	146	12.7	1.†	982	242	33039	4731	33912

### Mouse dose range-finding efficacy

**Fig 4:**
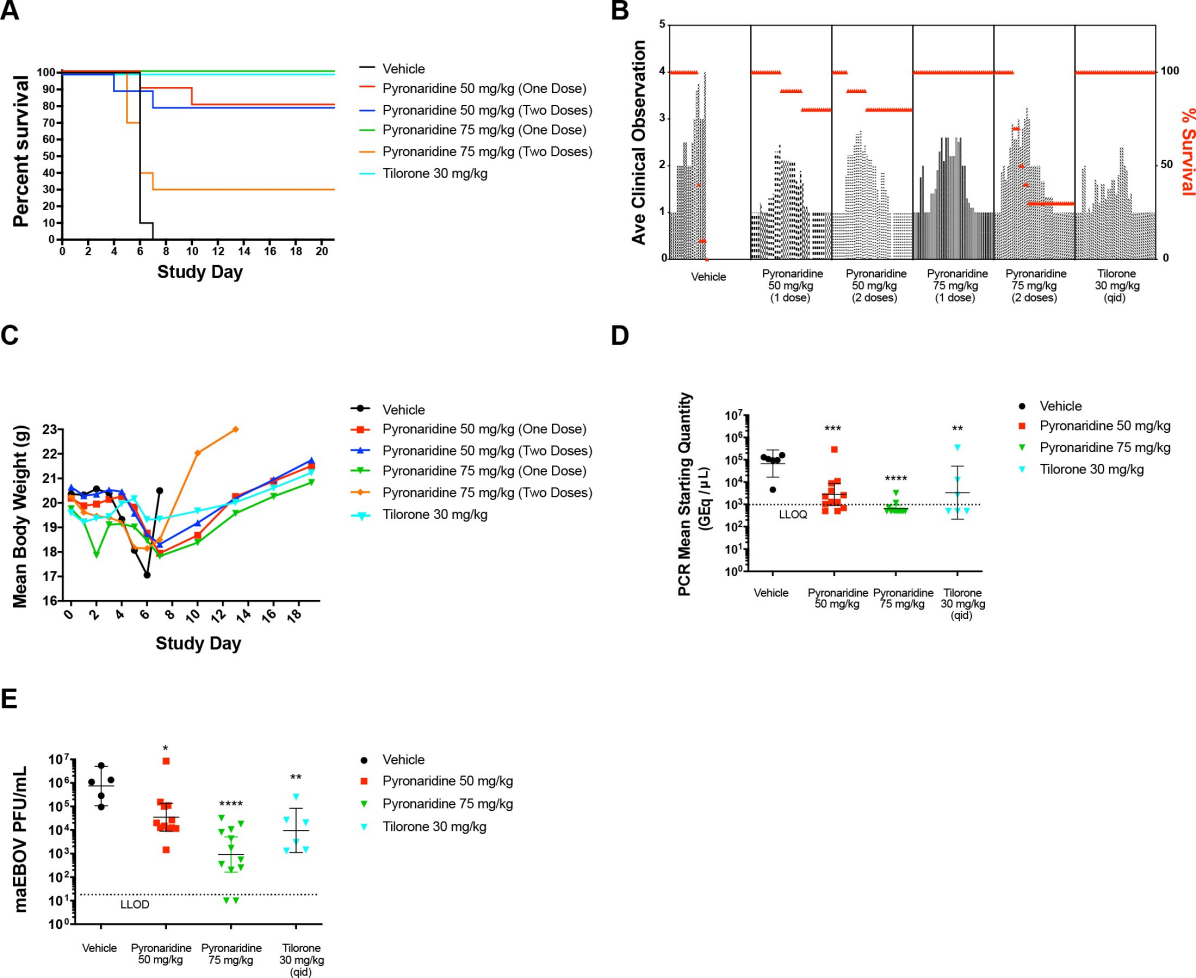
maEBOV efficacy data. (A) The survival curves between pyronaridine 50 mg/kg (1 or 2 doses), pyronaridine 75 mg/kg (1 dose) and tilorone 30 mg/kg were not statistically significantly different using a log-rank (Mantel-Cox) test (p = 0.2268). (B) Mean clinical scoring results with overlaid percent survival. (C) Mean body weight results. (D) qRT-PCR measurement of viral RNA in sera (mice sacrificed on SD3). Dunnett’s test, **, Adjusted p = 0.0071, ***; Adjusted p = 0.0010, ****, Adjusted p < 0.0001, (E) Plaque assay for viable EBOV in sera (mice sacrificed on SD3) (Dunnett’s test: control vs pyronaridine 50 mg/kg, Adjusted P = 0.0424 (*); control vs pyronaridine 75 mg/kg, Adjusted P < 0.0001; control vs tilorone 30 mg/kg, Adjusted P = 0.0094 (**)). Statistical significance was calculated with log-transformed qPCR and plaque assay data using a Dunnett’s test with the vehicle designated as the control. PCR mean starting quantity and maEBOV viral load had LLOD/LLOQ of 1000 GEq/μl and 20 PFU/ml, respectively. Quantified values below these where set to 0.5 x LLOD/LLOQ. Bars and error-bars represents the geometric mean and 95% CI, respectively.

The efficacy of pyronaridine against mouse-adapted EBOV (maEBOV) was evaluated in mice across a range of concentrations and several doses. BALB/c mice were randomly assigned into a group of 8 male and 8 female and were administered either pyronaridine, tilorone (positive control; 30 mg/kg q.d. (study day (SD) 0 –SD7)), or vehicle via i.p. injection. The virus challenge day was defined as SD0. Pyronaridine was dosed at 50 or 75 mg/kg i.p. either on SD0 or SD0 and SD4, with the initial treatment dose given ~1 h post-challenge. The optimal dosing of tilorone was previously determined to be 30 mg/kg per injection [37], administered q.d. on SD 0–7. Fig 4A shows that none of the challenged vehicle control mice survived past SD7 and 100% of the challenged tilorone control mice survived for the duration of this study. Both groups of 50 mg/kg pyronaridine treated (one dose or two doses) mice had identical final survival rates of 80%. The 75 mg/kg groups had substantially different survival rates of 100% (one dose, SD0) and 30% (two doses, SD0 and SD4) (Fig 4A). The two-dose group’s decreased survival rates may have resulted from the toxicity of multiple doses of pyronaridine via i.p. administration. All groups showed an increase in the clinical scoring after challenge, but survivors returned to normal by the end of the study (Fig 4B). Likewise, mean body weight loss was seen in all groups after challenge, but survivors returned to pre-challenge body weight or above prior to study finalization (Fig 4C).

On study day 3, 6 mice (3 male, 3 female) were euthanized from each group, serum was harvested, and viremia measurements were ascertained via plaque assay (S5 Table) and quantitative RT-PCR (Table 3). Fig 4D illustrates the level of viral RNA in the serum on day 3 after challenge. Vehicle control mice had a mean of 6.73 x 10^4^ GEq/mL and the 50 mg/kg pyronaridine group showed a significant (Dunnett’s test, ***; Adjusted p = 0.0010), 20-fold reduction in viral RNA 2.75 x 10^3^ GEq/mL while 75 mg/kg of pyronaridine had a more pronounced effect on the levels of viral RNA with a mean level of 6.53 x 10^2^ GEq/mL (Dunnett’s test, ****, Adjusted p < 0.0001). Ten of the twelve samples from this group were below the LLOQ (1 x 10^3^ GEq/ml). Tilorone also caused a similar statistically significant reduction in viral RNA with a mean of 3.37 x 10^3^ GEq/mL (Dunnett’s test; **, Adjusted p = 0.0071) with 3 of the 6 samples below the LLOQ, where the means represent geometric means.

**Table 3:** Quantitative RT-PCR and Plaque Assay Results for Animals euthanized on SD3.

SD3	Group	Vehicle	Pyronaridine 50 mg/kg	Pyronaridine 75 mg/kg	Tilorone (30 mg/kg)
	N	6	12	12	6
RT-PCR (GEq/uL)	Geometric Mean	6.74E+04	2.75E+03	6.53E+02	3.37E+03
95% CI	Lower 95% CI	1.66E+04	8.86E+02	4.56E+02	2.17E+02
	Upper 95% CI	2.73E+05	8.56E+03	9.35E+02	5.22E+04
Plaque (PFU/mL)	Geometric Mean	7.39E+05	3.51E+04	9.13E+02	9.57E+03
95% CI	Lower 95% CI	1.07E+05	8.97E+03	1.63E+02	1.11E+03
	Upper 95% CI	5.10E+06	1.37E+05	5.12E+03	8.29E+04

We also measured the amount of viable virus (Fig 4E) in the serum by plaque assay (S5 Table). Mice that received vehicle only had a mean of 7.40 x 10^5^ PFU/mL. Similar to the qPCR assay, the 50 mg/kg pyronaridine group gave a 20-fold decrease in virus titer to 3.49 x 10^4^ PFU/mL and a more dramatic decrease in the in the 75 mg/kg group (9.14 x 10^2^ PFU/mL) compared to the control group. There was also a significant reduction in the tilorone treated group (9.57 x 10^3^ PFU/mL; Fig 4E). All reductions from the vehicle group were statistically significant (Dunnett’s test: control vs pyronaridine 50 mg/kg, Adjusted P = 0.0424 (*); control vs pyronaridine 75 mg/kg, Adjusted P < 0.0001; control vs tilorone 30 mg/kg, Adjusted P = 0.0094 (**)). Viremia was quantified in the serum of mice that met the criterion for euthanasia. All mice from which samples were obtained were positive for viral RNA and viable virus (S5 Table).

### Mouse treatment window efficacy

**Fig 5:**
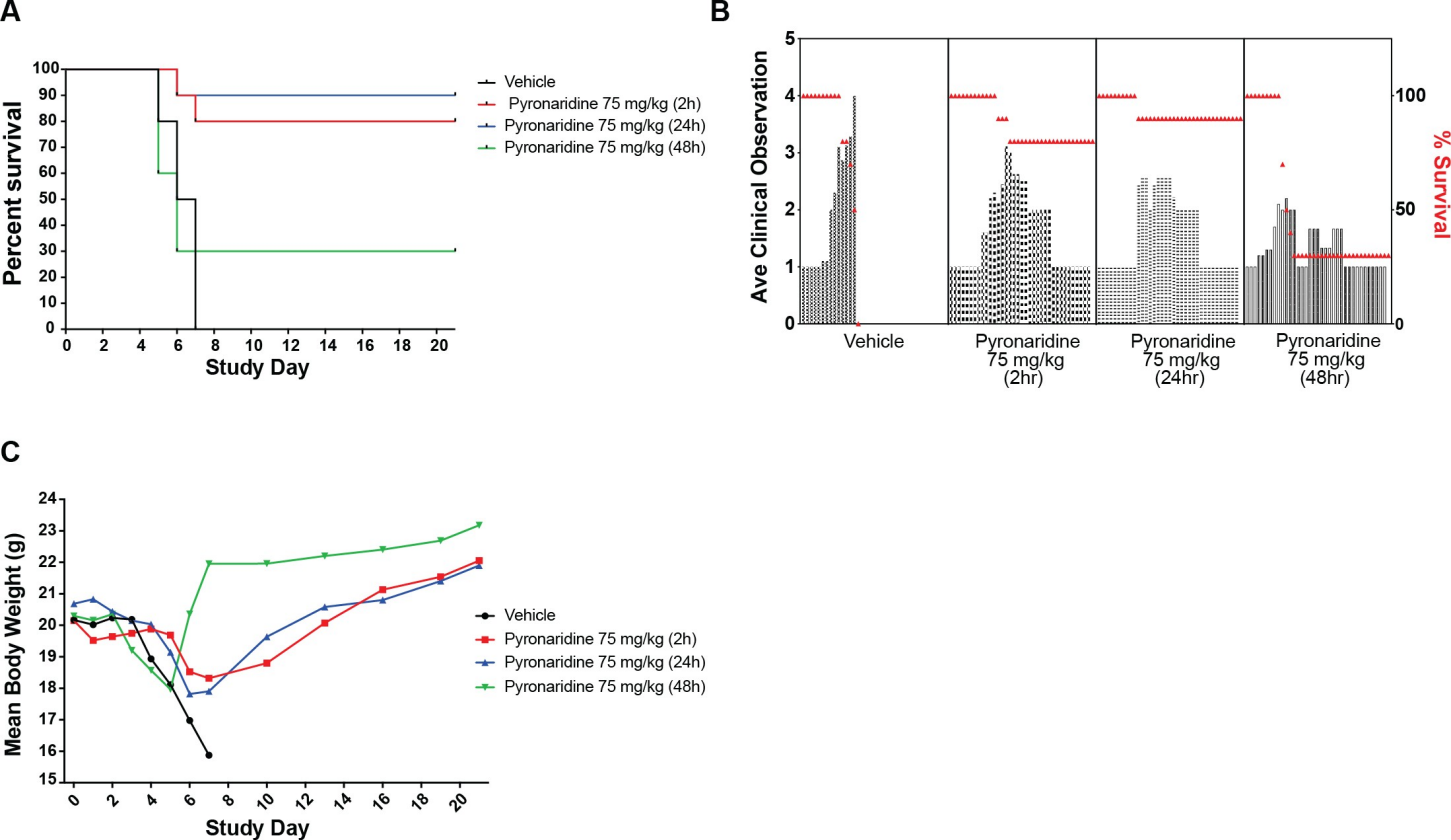
Mouse EBOV window study. (A) The survival curves between pyronaridine 75 mg/kg administered at 2 or 24h post infection were not statistically significantly different using a log-rank (Mantel-Cox) test (p = 0.5567), while pyronaridine administered at 48h was not statistically different than the vehicle (p = 0.7782). B) Mean clinical scoring results with overlaid percent survival. (C) Mean body weight results.

With the optimal single dose of 75 mg/kg, based on the dose range-finding study, we experimentally validated the window of efficacy of pyronaridine against maEBOV (Fig 5). The treatment was administered i.p. 2, 24, and 48 h post-challenge to groups of experimentally naïve BALB/c mice (n = 10, gender balanced). The window for i.p. dosing in mice was determined and we found treatment 2h or 24h post-challenge resulted in 80 and 90% survival, respectively. The survival decreased to 30% when treatment was delayed until 48 h after challenge (Fig 5A). Similar to the dose ranging study, all groups showed a mean increase in clinical scores (Fig 5B) and a mean decrease in body weights following challenge (Fig 5C). Survivors returned to prestudy body weight values by the end of the study.

### Cytokine and chemokine analysis

**Fig 6:**
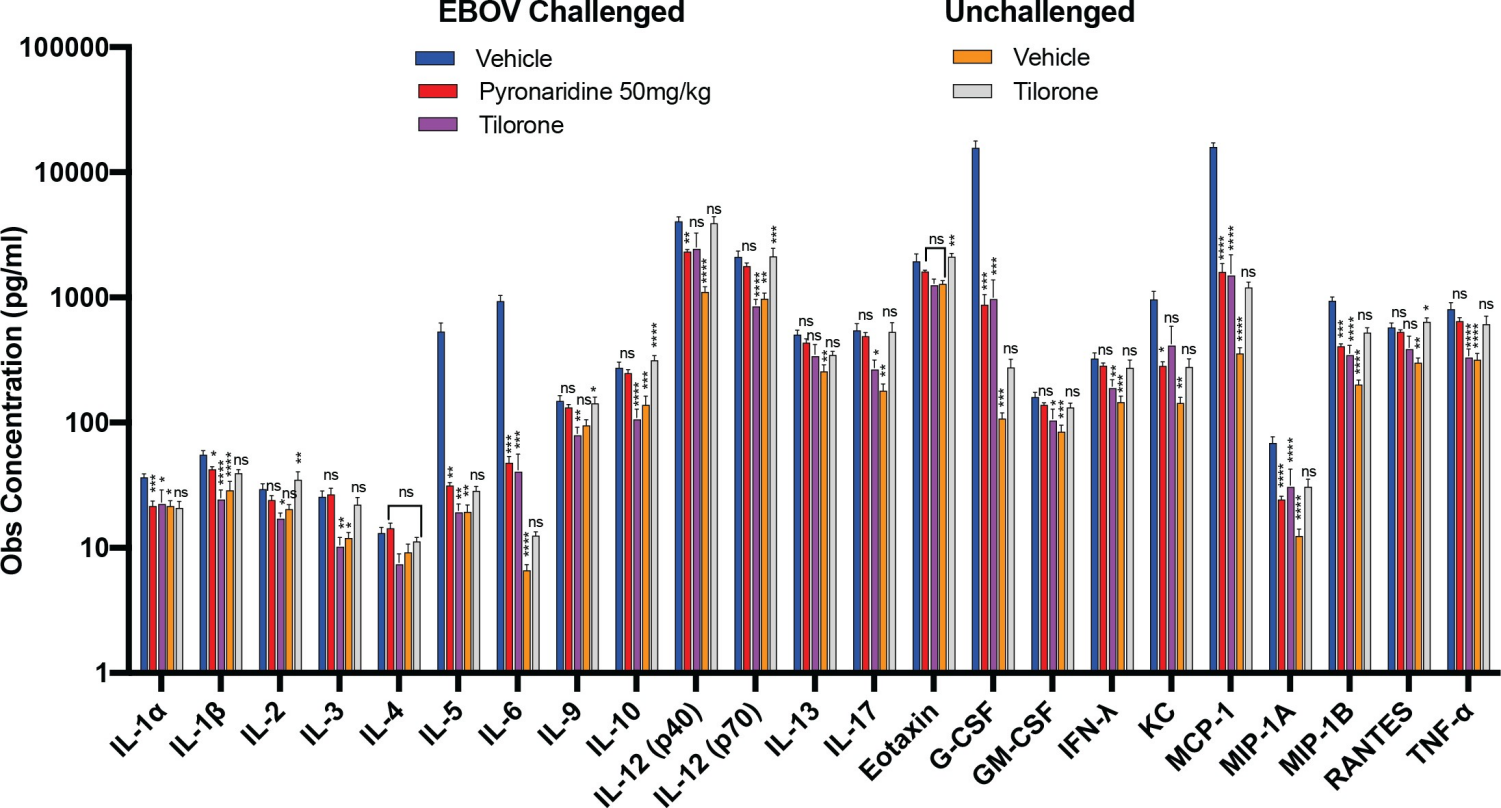
The observed concentration of each cytokine/chemokine was from either challenged (EBOV) or unchallenged (UN) mice, with the serum from each euthanized mouse run in duplicate. Each challenged vehicle (EBOV) and tilorone (EBOV) group is comprised of 6 mice and the unchallenged, vehicle and tilorone groups each had 4 mice each. Each bar represent the mean of the group with error bars representing the SEM. The statistical significance (0.0021< * ≤ 0.0332, 0.0002< ** ≤ 0.0021, 0.0002< *** ≤ 0.0001, **** < 0.0001) of the difference between the EBOV challenged vehicle group (negative control) and the test or control article (pyronaridine or tilorone) groups is displayed above each bar. The identifier is centered on its respective treated group. Significance was determined by using a Tukey or Dunnett’s T3 test.

The cytokine and chemokine analysis consisted of Eotaxin, G-CSF, GM-CSF, IFN-γ, IL-1α, IL-1β, IL-2, IL-3, IL-6, IL-9, IL-10, IL-12 (p40), IL-12 (p70), IL-13, IL-17A, KC, MCP-1, MIP-1a, MIP-1b, RANTES and TNF-α. Many of these exhibited a statistically significant increase over vehicle with tilorone alone (Eotaxin (Adjusted P = 0.0099, **), IL-2 (Adjusted P = 0.0409, *), IL-4, IL-5, IL-6 (Adjusted P = 0.0083, **), IL-10 (Adjusted P < 0.0001, ****), IL-12 (p40) (Adjusted P = 0.0145, *), IL-12 (p70) (Adjusted P = 0.0068, **), IL-17A (Adjusted P = 0.0067, **), MCP-1 (Adjusted P = 0.0038, **), MIP-1b (Adjusted P = 0.0032, **), RANTES (Adjusted P = 0.0006, ***) in unchallenged mice (S5 Fig), but this was not mirrored with pyronaridine (S6 Fig). In maEBOV challenged-mice a strong immune response occurred with significant increases over the unchallenged vehicle for every cytokine and chemokine tested (Fig 6). Similar increases in cytokines and chemokines were seen in tilorone- and pyronaridine-treated maEBOV challenged mice as compared to unchallenged mice, even with drastically reduced viral loads in the test and positive control article-treated groups (Fig 6). In many cases the maEBOV challenged mice treated with tilorone or pyronaridine had a similar response to the maEBOV vehicle-treated group (not statistically different, Fig 6), irrespective of the large difference in the viral loads. Surprisingly, this is much more common with pyronaridine and not tilorone treated-mice, suggesting that the immune response is elevated by pyronaridine more often than with the known immunomodulator tilorone (Fig 6 and S6 Fig).

## Discussion

Currently there is no FDA or EMA approved therapeutic for use against EBOV which represents a significant gap in our readiness for outbreaks similar to the pandemic seen in Africa, which led to over 11,000 deaths [49]. The current outbreak in the Democratic Republic of the Congo has killed 2152 people and this points to the need for drugs even when there are vaccines available undergoing clinical trials. Recently it has been found that some survivors of EBOV can retain detectable virus for more than 18 months post initial infection, increasing the chances for the spread of the virus [50]. Because of global travel, health workers can potentially carry this virus outside of Africa, although this is rare. It is hence important that a potential treatment is developed that could protect health professionals when exposed to the virus. During the previous EBOV outbreak in Africa thousands of US troops provided critical support providing facilities and medical support staff [51]. To date there have been relatively few studies describing potential small molecule inhibitors of EBOV [4, 52]. The only small molecule drugs that have undergone human clinical testing are antivirals targeting the Ebola RNA polymerase (Favipiravir [53], GS-5734 (Remdesivir, an antiviral drug) [54] and Galidesivir [54] as well as drug combinations of existing FDA-approved drugs (GBV-006)). Several compounds are in development at the preclinical stage targeting viral entry, host pathways and innate immunity pathways [28, 55]. The recent survival results with the monoclonal antibodies REGN-EB3 [11],and mAb114 [10] appear promising [12] but to date none of these potential treatments are FDA or EMA approved. Despite this, there still remains a significant need for clinically proven anti-EBOV therapeutics and prophylactic agents.

Tilorone, identified in our previous work [37], and pyronaridine have efficacy for protecting mice from maEBOV disease (Fig 4A). Pyronaridine tetraphosphate is a benzonaphthyridine derivative that was first synthesized in 1970 at the Institute of Chinese Parasitic Diseases [56, 57]. It is a potent [58–60] antimalarial that has some similarity in structure to chloroquine, but with several advantages with respect to its side effects and pharmacokinetics. For example, the LD_50_ for pyronaridine in mice is reported as 1342 mg/kg, indicating relatively low toxicity (animals did not show behavioral abnormalities or neurotoxicity) as compared to chloroquine (LD_50_ 654 mg/kg) [17]. We are therefore currently evaluating the use of this compound against several pathogens [29, 31, 61]. It has been widely used in China for more than 30 years as a single agent against *Plasmodium falciparum*, the major causative parasite of malaria (*in vitro* EC_50_ = 13.5 nM [59]), and in multi-drug, artemisinin-based combination therapies (ACTs). One of the ACTs is Pyramax, which is a fixed 3:1 ratio combination of pyronaridine and artesunate, respectively. The use of Pyramax has received Positive Opinion by the European Medicines Agency [31] and in 2017 this combination was included on the WHO’s Model List of Essential Medicines (EML) and Model List of Essential Medicines for Children (EMLc) for the treatment of malaria. While we recently found pyronaridine had potent activity against EBOV *in vitro* [29, 30], it has also shown activity against the erythrocyte-infecting *Babesia spp* parasite [62] as well as inhibition against *Trypanosoma cruzi*, the causative agent of Chagas disease [63].

It is compelling to suggest that the reason for effective inhibition of EBOV by pyronaridine in HeLa and not Vero cells is simply due to the lack of IFN signaling. There are however many different potential reasons for these differences. One possibility is that the viral entry mechanism is cell-dependent. There are multiple examples of this between these two cell lines. For example, Herpes Simplex Virus (HSV) has been shown to have a cell-type dependent entry mechanism that differs between HeLa and Vero cells. While the virus is endocytosed in both cell lines similarly, in HeLa cells, lysosomotrophic agents block HSV infection in a dose-dependent manner but they have no inhibitory effects on HSV entry in Vero cells [64]. Tilorone is an example of a known lysosomotrophic compound [65] as is the structurally related quinacrine [66]. Interestingly, chloroquine is unable to inhibit HSV infection in Vero Cells [67] but does inhibit in HeLa cells [68]. Vaccinia virus also exhibits cell-type-dependent entry, with heparin strongly inhibiting entry into HeLa cells, but not inhibiting entry into Vero cells [69]. A final example is Chikungunya Virus which has a higher binding and infectivity rate in Vero versus HeLa cells [70]. Such cell specific effects have therefore been widely reported.

There is also evidence that EBOV may have variable entry pathways that are cell-type dependent. EBOV particles appear to be internalized in a clathrin-dependent manner in in HeLa cells but not in Vero cells [71]. Previously it had been shown that inhibition varies between HeLa and Vero EBOV-infected cells for the Acid sphingomyelinase (ASMase) inhibitors imipramine and desipramine. These were both less potent in Vero cells with EC_50’_s 2 to 3 times higher than those found for HeLa cells. Imipramine was also shown to significantly inhibit the entry step of EBOV infection in HeLa cells, but this was not confirmed in Vero cells [72]. This finding confirms that there can be cell-specific variations in EBOV inhibition that are likely independent of IFN signaling.

Since pyronaridine has an EC_50_ of between 420 nM—1.14 μM against EBOV in HeLa cells and an approximate CC_50_ of 3 μM in Vero cells, a similar variation would require pyronaridine to be at toxic levels in Vero cells to see inhibition. Therefore, there is not sufficient evidence to suggest that the mechanism of action of pyronaridine would be different between cell lines. Unfortunately, since the SI of pyronaridine is ~3 in HeLa cells, IFN-knockout or knockdown will likely mask if IFN signaling is involved due to the undesired off-target effects of siRNA or CRISPR [73], making these difficult to test. Additionally, since there is no reason to believe that the lack of IFN expression in Vero cells is related to the cytotoxicity of pyronaridine in these cells, reconstitution of IFN signaling is also not an option.

Independent data suggest that pyronaridine may be an immune modulator. Based on the cytokine profiling of peripheral blood mononuclear cells (PBMC) pyronaridine has been shown to induce several changes in the profile of intracytoplasmic cytokines by enhancing IL-4 production of monocytes while reducing TNF-α in CD8+ T cells and IL-10 and IL-4 in B-cells [74]. In addition, there was a higher frequency of CD14 and CD4 cells expressing TNF-α and IL-10 as well as an increased frequency of CD4 cells expressing Il-2 and INF- γ [74]. Analysis of our *in vivo* data shows that in uninfected mice pyronaridine administered i.p. (50 or 75 mg/kg) did not elicit a statistically significant increase (Tukey’s HSD or Dunnett’s T3 test) in any of the cytokine/chemokines tested over vehicle or naïve controls, suggesting that responses to pyronaridine may be more complex in an EBOV infected animal model.

Tilorone has been known for decades to be an immunomodulator both *in vivo* [75, 76] and *in vitro* [77], but this modulation had been previously understood to be through the stimulation of type I IFN expression. Our data suggests that tilorone may also have a more complex action by activating many different pathways associated with the immune system, with a statistically significant increase in observed protein concentrations in tilorone-treated unchallenged mice over both vehicle-treated and naïve mice in 9 of the 23 chemokines/cytokines tested (S5 Fig). Tilorone has also been shown to increase *in vivo* secretion of IL-6, TNF-a and IL-12 in macrophages from naïve BALB/c mice [78]. The activation of multiple aspects of the immune system by tilorone is not surprising given its effectiveness in treating maEBOV [37]. In contrast, it has been previously shown *in vivo* in mice (IFN-ß) and cynomolgus monkeys (IFN- α/ß) that type I IFN given directly alone is unable to protect against EBOV [79, 80]. In the current study IFN- α/ß were not at detectable levels in unchallenged mice administered tilorone q.d. for 3 days at 30 mg/kg, so it is unknown whether this dosing directly affected IFN production as compared to naïve or vehicle-injected mice. Analysis of the published work on the EBOV immune response illustrates the complexity in this disease (S1 Supplemental text and references).

Comparison of the observed concentrations of each of the cytokine and chemokines tested shows that the maEBOV-challenged mice administered the vehicle are in most cases significantly higher (Tukey’s HSD test) than their unchallenged or naïve counterparts. Exceptions are IL-2, IL-4, IL-9, and Eotaxin. Many of the cytokines and chemokines were also significantly increased in the unchallenged, tilorone-administered group over vehicle and naïve groups (S5 Fig), suggesting that there may be a correlation between the immune response and tilorone’s mechanism of action. Unchallenged mice injected with tilorone vs vehicle and naïve groups showed significantly higher levels of IL-6, IL-10, IL-12 (p40), IL-12 (p70), IL-17, Eotaxin, MCP-1, MIP-1B and RANTES. IL-10, IL-12 (p40), MCP-1, MIP-1B and RANTES. In multiple cases the unchallenged tilorone mice had a significantly higher response for many of the cytokines and chemokines as compared with the maEBOV-challenged group administered tilorone. This is the case for IL-2, IL-9, IL-10, IL-12 (p70), Eotaxin and RANTES. EBOV has only been shown to be able to modulate type I IFN production and signaling, so it would be expected that only those cytokines and chemokines directly linked to pathways downstream of IFN signaling would be able to be suppressed by the virus. Based on previous *in vitro* work, IFN signaling can only induce IL-10 and IL-6 expression and suppress IL-12 and IL-1 expression in cells [81–83], challenging the notion that IFN signaling alone is suppressed by EBOV.

The quantified viral load from the plaque and q-PCR assays suggests that the EBOV-challenged vehicle group had at least an order of magnitude reduction (PFU/ml and GEq/μl) in the treated groups (Fig 4D and 4E), so differences in cytokines and chemokine levels between these groups may be attributed to the amount of viremia present. Statistically (Tukey’s HSD test) there are no differences in the viral loads between the pyronaridine 50 mg/kg and tilorone-treated groups on study day 3, therefore any differences between pyronaridine 50mg/kg and tilorone treated groups may point to a variation in mechanism between these two drugs. It is noted that there is a statistically significant difference between the pyronaridine 75 mg/kg and 50 mg/kg groups in the plaque assay, with respective geometric means of 9.13 x 10^2^ and 3.49 x 10^4^ PFU/ml (Tukey’s HSD test). Since there is strong evidence from previous studies that showed tilorone is an immunomodulator [75–77], this indicates that the mechanism of action against maEBOV is likely to be at least partly due to the host immune response. We now show for the first time an increase in nearly all of the cytokine and chemokines tested in the tilorone-treated, unchallenged group (S5 Fig). From our findings in this (Fig 4A) and a previous study, tilorone has 100% efficacy against maEBOV [37] which indicates immune modulation is at least partially responsible for tilorone’s efficacy. Pyronaridine demonstrates bioactivity against unrelated pathogens [29, 31, 61–63], pointing to a mechanism of action that may also involve a host effect (or common targets). Analysis of the current cytokine and chemokine data demonstrates pyronaridine does not illicit an immune response in unchallenged mice (not statistically different from both vehicle/naïve using Tukey’s HSD or Dunnett’s T3 test), but in maEBOV-challenged mice the response is equivalent to or increased from those treated with tilorone (Fig 6). Pyronaridine may have an effect on the levels of IFN-α/β, but the concentrations in the groups injected with vehicle and pyronaridine were below the level of detection for the assay used. Even with the known IFN-inducer tilorone IFN-α/β levels were also below the LLOD on SD3, suggesting that induction of IFN below the LLOD of the assay used may still be sufficient for viral protection. The pyronaridine response to EBOV exceeds the tilorone-treated group in many cases and is statistically equivalent to the control (Fig 6) that has at minimum an order of magnitude higher viremia levels. Pyronaridine and tilorone may therefore mitigate EBOV propagation by activating the immune system in a similar manner, and in some respects both these molecules appear to act as adjuvants.

Pyronaridine has been shown to have an extended half-life in humans, with a half-life elimination in whole blood of approximately 17 d [84] and 33.5 d for the total radioactive half-life [85] which compares favorably with our observations in mice in this study (Fig 3). A mass balance study in healthy volunteers indicated nine primary and four secondary metabolites of pyronaridine [85] and recombinant CYP studies suggested that these metabolites were generated by cytochrome P450s CYP1A2, CYP2D6, and CYP3A4 [31].

A prior study of pyronaridine tested the efficacy against *T*. *cruzi* challenged mice at similar concentrations (50 mg/kg), but twice a day for 4 days [4], resulted in an efficacy rate of 85%. Considering the long half-life of pyronaridine in mice shown in the current study, the blood concentration in the mice from the previous study would have been much higher than in the current study. The current data suggests that the 2-dose 75 mg/kg mice low survival rate (Fig 4A) may be due to toxicity of the multiple doses, which could be specific to the disease state created by maEBOV. The potential for pyronaridine to be a practical treatment regimen against EBOV is demonstrated as the effective dose in mouse is 75 mg/kg, which can be scaled to a human dose based on body surface areas by dividing by 12.3 [86] which equates to 6.1mg/kg in human. Taking an average human of 60kg, this represents a dose of 366mg. One study has already reported pharmacokinetics of pyronaridine in humans after oral dose of 400 mg with a t_1/2_ = 241 hours and volume of distribution 41.2 L/kg [47].

In summary, the observations demonstrating efficacy of pyronaridine against maEBOV and activation of the immune system during infection indicate that it warrants further study for repurposing against this and other filoviruses. It would be highly advantageous for testing as an oral regimen for those with an active EBOV infection.

## Supporting information

S1 Table NIAID *in vitro* virus testing of pyronaridine. (DOCX)

S2 Table Final concentrations (μM) of tilorone and pyronaridine used for the *in vitro* synergy study (checkerboard assay). (DOCX)

S3 Table Metabolite profile of pyronaridine in Mouse liver microsomes. (DOCX)

S4 Table Maximum tolerated dose data for pyronaridine. (DOCX)

S5 Table Quantitative RT-PCR and plaque assay results for animals that met euthanasia criteria. (DOCX)

S1 Fig The BRAID analysis calculates synergy by fitting data to a seven-variable function. The variable κ represents a quantitative synergy value. κ < 0 implies antagonism, κ  =  0 implies additivity, and κ > 0 implies synergy. The other variables are E_0_, the estimated effect when neither drug is present; E_t_, the maximal effects of either drug alone; ID_M,A_ and ID_M,B_ concentrations representing the EC_50_ of either drug alone; and n_a_ and n_b_, are the Hill equation parameters representing the sigmoidicity of both drugs’ dose response curves. A) The BRAID effect is the plot of the best BRAID fit. The BRAID error is the difference between the smoothed data and the best BRAID fit. The higher the R^2^ the better the fit the BRAID fit is to the raw, smoothed data. B) The potentiation of drugs is the interpolated effect curves for the drugs in combinations using the best BRAID fit equation. Data represents a checkboard assay with pyronaridine and tilorone at various combined concentrations (Fixed pyronaridine/tilorone concentrations of 0.012, 0.024, 0.049, 0.098, 0.195, 0.391, 0.781, 1.562, 3.125, 6.25, 12.5, or 25 μM) in HeLa cells. A calculated κ  = 0.488 (- 0.543–8.18) suggested that these compounds are synergistic to each other for the inhibition of EBOV in HeLa cells. The BRAID analysis shows potentiation of the EC_50_ of tilorone by pyronaridine, but due to toxicity the potentiation of pyronaridine by tilorone could not be accurately analyzed.
(TIF)

S2 Fig Metabolite ID data. (TIF)

S3 Fig TIC Chromatogram* of Incubation Sample of Pyronaridine (right panel: T = 0 min and left panel: 120 min). * For profiling only, each peak may have different ionization efficiency. *, in the incubation sample at 60 or 120 min, the abundance of potential metabolites (M1-M5) are normalized to that of parent drug (100%) based on the peak height.
(TIF)

S4 Fig Mass Spectrum and Proposed Structure of Pyronaridine Important Metabolites in Mouse liver microsomes. (TIF)

S5 Fig The observed concentration of each cytokine/chemokine was from unchallenged mice, with the serum from each euthanized mouse run in duplicate. Each group was unchallenged (vehicle, naïve and tilorone) with each group comprised of 4 mice. Bars represent the mean and the error bars represent the SEM. Stars represent the significance of the difference from the unchallenged, tilorone-treated group (Tukey test). (0.0021< * ≤ 0.0332, 0.0002< ** ≤ 0.0021, 0.0002< *** ≤ 0.0001, **** < 0.0001).
(TIFF)

S6 Fig Comprehensive immunological data from SD3 mice. The observed concentration of each cytokine/chemokine was from either challenged (EBOV) or unchallenged (UN) mice, with the serum from each euthanized mouse run in duplicate. Each challenged vehicle (EBOV) and tilorone (EBOV) group comprised of 6 mice, while the pyronaridine 50 and 75 mg/kg (EBOV) groups had 12 mice each. The unchallenged groups each had 4 mice each. Error bars represent the SEM.
(TIFF)

S1 Supplemental Text and References (DOCX)
