# Supplementary material for: Repurposing the antimalarial pyronaridine tetraphosphate to protect against Ebola virus infection
Source: PLoS Negl Trop Dis. 2019 Nov 21;13(11):e0007890. doi: 10.1371/journal.pntd.0007890 (PMC6894882; doi:10.1371/journal.pntd.0007890)
Supplement: S1 Table — (DOCX) [file pntd.0007890.s001.docx]

**S1 Table.** NIAID *in vitro* virus testing of pyronaridine.

|  | **Cell line** | **EC_50_ (µM)** | **EC_90_ (µM)** | **CC_50_ (µM)** | **SI_50_** | **SI_90_** |
| --- | --- | --- | --- | --- | --- | --- |
| Herpes Simplex Virus 1 | HFF | >6 | >6 | 6.52 | <1 | <1 |
| Vaccinia Virus | HFF | >1.20 | >1.20 | 4.02 | <3 | <3 |
| Chikungunya virus | Vero 76 | 3.2 |  | 3.2 | 1 |  |
| Dengue Virus 2 | Vero 76 | 3.2 |  | 3.2 | 1 |  |
| Ebola virus | Vero 76 | >1.3 |  | 1.3 | 0 |  |
| Influenza A virus H1N1 | MDCK | 3.2 |  | 3.2 | 1 |  |
| MERS coronavirus | Vero 76 | 3.2 |  | 3.2 | 1 |  |
| Poliovirus 3 | Vero 76 | 3.2 |  | 3.2 | 1 |  |
| Respiratory synactial virus | MA-104 | >7.5 |  | 7.5 | 0 |  |
| Rift Valley fever virus | Vero 76 | 3.2 |  | 3.2 | 1 |  |
| Tacaribe virus | Vero | >3.2 |  | 3.2 | 0 |  |
| Venezuelian equine encephalitis virus | Vero 76 | 1.8 |  | 3.2 | 1.8 |  |
| West Nile virus | Vero 76 | >24 |  | 24 | 0 |  |
| Yellow Fever virus | Vero 76 | 3.2 |  | 3.2 | 1 |  |
| Zika virus | Vero 76 | 3.2 |  | 3.2 | 1 |  |
| Zika virus | Huh7 | 3.2 |  | 3.2 | 1 |  |
| Norovirus | HG23 | 3.5 | 9.7 | >100 | 29 | 10 |
| Murine norovirus | RAW267.4 | >40 | >40 | >40 | 1 | 1 |
| Human cytomegalovirus | HFF | >1.2 | >1.2 | 3.17 | <3 | <3 |
| Hepatitis C virus | Huh7 | 0.78 | 1.71 | 0.89 | 1 | <1 |
| Hepatitis B virus | HepG2 2.2.15 | 4.52 | >100 | 3.54 | <1 | <1 |
