# Supplementary material for: Repurposing the antimalarial pyronaridine tetraphosphate to protect against Ebola virus infection
Source: PLoS Negl Trop Dis. 2019 Nov 21;13(11):e0007890. doi: 10.1371/journal.pntd.0007890 (PMC6894882; doi:10.1371/journal.pntd.0007890)
Supplement: S2 Table — (DOCX) [file pntd.0007890.s002.docx]

**S2 Table.** Final concentrations (µM) of tilorone and pyronaridine used for the *in vitro* synergy study (checkerboard assay).

| 1 | 2 | | 3 | | 4 | | 5 | | 6 | | 7 | | 8 | | 9 | | 10 | | 11 | | 12 | |  |  |
| --- | --- | --- | --- | --- | --- | --- | --- | --- | --- | --- | --- | --- | --- | --- | --- | --- | --- | --- | --- | --- | --- | --- | --- | --- |
| a | Tilorone-25  Pyronaridine-25 | | Tilorone-12.5  Pyronaridine-25 | | Tilorone-6.25  Pyronaridine-25 | | Tilorone-3.13  Pyronaridine-25 | | Tilorone-1.56  Pyronaridine-25 | | Tilorone-0.78  Pyronaridine-25 | | Tilorone-0.39  Pyronaridine-25 | | Tilorone-0.2  Pyronaridine-25 | | Tilorone-0.1  Pyronaridine-25 | | Tilorone-0.05  Pyronaridine-25 | | Tilorone-0.02  Pyronaridine-25 | | Tilorone-0.01  Pyronaridine-25 | |
| b | Tilorone-25  Pyronaridine-12.5 | | Tilorone-12.5  Pyronaridine-12.5 | | Tilorone-6.25  Pyronaridine-12.5 | | Tilorone-3.13  Pyronaridine-12.5 | | Tilorone-1.56  Pyronaridine-12.5 | | Tilorone-0.78  Pyronaridine-12.5 | | Tilorone-0.39  Pyronaridine-12.5 | | Tilorone-0.2  Pyronaridine-12.5 | | Tilorone-0.1  Pyronaridine-12.5 | | Tilorone-0.05  Pyronaridine-12.5 | | Tilorone-0.02  Pyronaridine-12.5 | | Tilorone-0.01  Pyronaridine-12.5 | |
| c | Tilorone-25  Pyronaridine-6.25 | | Tilorone-12.5  Pyronaridine-6.25 | | Tilorone-6.25  Pyronaridine-6.25 | | Tilorone-3.13  Pyronaridine-6.25 | | Tilorone-1.56  Pyronaridine-6.25 | | Tilorone-0.78  Pyronaridine-6.25 | | Tilorone-0.39  Pyronaridine-6.25 | | Tilorone-0.2  Pyronaridine-6.25 | | Tilorone-0.1  Pyronaridine-6.25 | | Tilorone-0.05  Pyronaridine-6.25 | | Tilorone-0.02  Pyronaridine-6.25 | | Tilorone-0.01  Pyronaridine-6.25 | |
| d | Tilorone-25  Pyronaridine-3.13 | | Tilorone-12.5  Pyronaridine-3.13 | | Tilorone-6.25  Pyronaridine-3.13 | | Tilorone-3.13  Pyronaridine-3.13 | | Tilorone-1.56  Pyronaridine-3.13 | | Tilorone-0.78  Pyronaridine-3.13 | | Tilorone-0.39  Pyronaridine-3.13 | | Tilorone-0.2  Pyronaridine-3.13 | | Tilorone-0.1  Pyronaridine-3.13 | | Tilorone-0.05  Pyronaridine-3.13 | | Tilorone-0.02  Pyronaridine-3.13 | | Tilorone-0.01  Pyronaridine-3.13 | |
| e | Tilorone-25  Pyronaridine-1.56 | | Tilorone-12.5  Pyronaridine-1.56 | | Tilorone-6.25  Pyronaridine-1.56 | | Tilorone-3.13  Pyronaridine-1.56 | | Tilorone-1.56  Pyronaridine-1.56 | | Tilorone-0.78  Pyronaridine-1.56 | | Tilorone-0.39  Pyronaridine-1.56 | | Tilorone-0.2  Pyronaridine-1.56 | | Tilorone-0.1  Pyronaridine-1.56 | | Tilorone-0.05  Pyronaridine-1.56 | | Tilorone-0.02  Pyronaridine-1.56 | | Tilorone-0.01  Pyronaridine-1.56 | |
| f | Tilorone-25  Pyronaridine-0.78 | | Tilorone-12.5  Pyronaridine-0.78 | | Tilorone-6.25  Pyronaridine-0.78 | | Tilorone-3.13  Pyronaridine-0.78 | | Tilorone-1.56  Pyronaridine-0.78 | | Tilorone-0.78  Pyronaridine-0.78 | | Tilorone-0.39  Pyronaridine-0.78 | | Tilorone-0.2  Pyronaridine-0.78 | | Tilorone-0.1  Pyronaridine-0.78 | | Tilorone-0.05  Pyronaridine-0.78 | | Tilorone-0.02  Pyronaridine-0.78 | | Tilorone-0.01  Pyronaridine-0.78 | |
| g | Tilorone-25  Pyronaridine-0.39 | | Tilorone-12.5  Pyronaridine-0.39 | | Tilorone-6.25  Pyronaridine-0.39 | | Tilorone-3.13  Pyronaridine-0.39 | | Tilorone-1.56  Pyronaridine-0.39 | | Tilorone-0.78  Pyronaridine-0.39 | | Tilorone-0.39  Pyronaridine-0.39 | | Tilorone-0.2  Pyronaridine-0.39 | | Tilorone-0.1  Pyronaridine-0.39 | | Tilorone-0.05  Pyronaridine-0.39 | | Tilorone-0.02  Pyronaridine-0.39 | | Tilorone-0.01  Pyronaridine-0.39 | |
| h | Tilorone-25  Pyronaridine-0.2 | | Tilorone-12.5  Pyronaridine-0.2 | | Tilorone-6.25  Pyronaridine-0.2 | | Tilorone-3.13  Pyronaridine-0.2 | | Tilorone-1.56  Pyronaridine-0.2 | | Tilorone-0.78  Pyronaridine-0.2 | | Tilorone-0.39  Pyronaridine-0.2 | | Tilorone-0.2  Pyronaridine-0.2 | | Tilorone-0.1  Pyronaridine-0.2 | | Tilorone-0.05  Pyronaridine-0.2 | | Tilorone-0.02  Pyronaridine-0.2 | | Tilorone-0.01  Pyronaridine-0.2 | |
| i | Tilorone-25  Pyronaridine-0.1 | | Tilorone-12.5  Pyronaridine-0.1 | | Tilorone-6.25  Pyronaridine-0.1 | | Tilorone-3.13  Pyronaridine-0.1 | | Tilorone-1.56  Pyronaridine-0.1 | | Tilorone-0.78  Pyronaridine-0.1 | | Tilorone-0.39  Pyronaridine-0.1 | | Tilorone-0.2  Pyronaridine-0.1 | | Tilorone-0.1  Pyronaridine-0.1 | | Tilorone-0.05  Pyronaridine-0.1 | | Tilorone-0.02  Pyronaridine-0.1 | | Tilorone-0.01  Pyronaridine-0.1 | |
| j | Tilorone-25  Pyronaridine-0.05 | | Tilorone-12.5  Pyronaridine-0.05 | | Tilorone-6.25  Pyronaridine-0.05 | | Tilorone-3.13  Pyronaridine-0.05 | | Tilorone-1.56  Pyronaridine-0.05 | | Tilorone-0.78  Pyronaridine-0.05 | | Tilorone-0.39  Pyronaridine-0.05 | | Tilorone-0.2  Pyronaridine-0.05 | | Tilorone-0.1  Pyronaridine-0.05 | | Tilorone-0.05  Pyronaridine-0.05 | | Tilorone-0.02  Pyronaridine-0.05 | | Tilorone-0.01  Pyronaridine-0.05 | |
| k | Tilorone-25  Pyronaridine-0.02 | | Tilorone-12.5  Pyronaridine-0.02 | | Tilorone-6.25  Pyronaridine-0.02 | | Tilorone-3.13  Pyronaridine-0.02 | | Tilorone-1.56  Pyronaridine-0.02 | | Tilorone-0.78  Pyronaridine-0.02 | | Tilorone-0.39  Pyronaridine-0.02 | | Tilorone-0.2  Pyronaridine-0.02 | | Tilorone-0.1  Pyronaridine-0.02 | | Tilorone-0.05  Pyronaridine-0.02 | | Tilorone-0.02  Pyronaridine-0.02 | | Tilorone-0.01  Pyronaridine-0.02 | |
| l | Tilorone-25  Pyronaridine-0.01 | | Tilorone-12.5  Pyronaridine-0.01 | | Tilorone-6.25  Pyronaridine-0.01 | | Tilorone-3.13  Pyronaridine-0.01 | | Tilorone-1.56  Pyronaridine-0.01 | | Tilorone-0.78  Pyronaridine-0.01 | | Tilorone-0.39  Pyronaridine-0.01 | | Tilorone-0.2  Pyronaridine-0.01 | | Tilorone-0.1  Pyronaridine-0.01 | | Tilorone-0.05  Pyronaridine-0.01 | | Tilorone-0.02  Pyronaridine-0.01 | | Tilorone-0.01  Pyronaridine-0.01 | |
|  |  |  |  |  |  |  |  |  |  |  |  |  |  |  |  |  |  |  |  |  |  |  |  |  |
