# Supplementary material for: Repurposing the antimalarial pyronaridine tetraphosphate to protect against Ebola virus infection
Source: PLoS Negl Trop Dis. 2019 Nov 21;13(11):e0007890. doi: 10.1371/journal.pntd.0007890 (PMC6894882; doi:10.1371/journal.pntd.0007890)
Supplement: S3 Table — (DOCX) [file pntd.0007890.s003.docx]

**S3 Table.** Metabolite Profile of Pyronaridine in Mouse liver microsomes.

| **Peak ID** | **RT (min)** | **Proposed metabolism** | **m/z (ESI+)** | **Mouse** |
| --- | --- | --- | --- | --- |
| Pyronaridine | 9.46 | Parent | 518.2 | 100%* |
| M1 | 12.3 | Oxidation | 534.3 | 10% |
| M2 | 12.4 | Oxidation | 534.3 | 5% |
| M3 | 12.8 | Hydrogenation+Oxidation | 536.3 | <5% |
| M4 | 14.4 | Oxidation | 532.3 | <5% |
| M5 | 15.0 | Di-Oxidation | 550.3 | <5% |
