# Supplementary material for: Repurposing the antimalarial pyronaridine tetraphosphate to protect against Ebola virus infection
Source: PLoS Negl Trop Dis. 2019 Nov 21;13(11):e0007890. doi: 10.1371/journal.pntd.0007890 (PMC6894882; doi:10.1371/journal.pntd.0007890)
Supplement: S4 Table — (DOCX) [file pntd.0007890.s004.docx]

**S4 Table.** Maximum tolerated dose data for pyronaridine.

| **Group** | **Dose (mg/kg)** | **No. of animals** | **Survival (no. of mice)** | **Clinical observations(s)** |
| --- | --- | --- | --- | --- |
| 1 | 10 | 3M / 3F | 6 of 6 | none |
| 2 | 50 | 3M / 3F | 6 of 6 | dehydration, hunched posture and ruffled fur |
| 3 | 100 | 3M / 3F | 6 of 6 | dehydration, hunched posture and ruffled fur |
| 4 | 300 | 3M / 3F | 0 of 6 | ataxia, dehydration, hunched posture,  ruffled fur and hypoactivity |
