# Supplementary material for: Repurposing the antimalarial pyronaridine tetraphosphate to protect against Ebola virus infection
Source: PLoS Negl Trop Dis. 2019 Nov 21;13(11):e0007890. doi: 10.1371/journal.pntd.0007890 (PMC6894882; doi:10.1371/journal.pntd.0007890)
Supplement: S5 Table — (DOCX) [file pntd.0007890.s005.docx]

**S5 Table.** Quantitative RT-PCR and Plaque Assay Results for Animals that Met Euthanasia Criteria

|  |  |  | **Pyronaridine 50 mg/kg** | | **Pyronaridine 75 mg/kg** | | **Tilorone (30 mg/kg)** |
| --- | --- | --- | --- | --- | --- | --- | --- |
|  | **Group** | **Vehicle** | **One Dose** | **Two Doses** | **One Dose** | **Two Doses** | **q.d.** |
|  | N | 9 | 1 | 1 | 0 | 5 | 0 |
| RT-PCR (GEq/uL) | Geometric Mean | 2.12E+05 | 2.57E+03 | 1.13E+05 | NA | 5.05E+04 | NA |
| 95% CI | Lower 95% CI | 4.95E+04 | NA | NA | NA | 1.65E+03 | NA |
|  | Upper 95% CI | 9.09E+05 | NA | NA | NA | 1.55E+06 | NA |
|  |  |  |  |  |  |  |  |
| Plaque (PFU/mL) | Geometric Mean | 8.09E+06 | 3.75E+07 | 1.50E+07 | NA | 7.88E+06 | NA |
| 95% CI | Lower 95% CI | 2.17E+06 | NA | NA | NA | 8.17E+04 | NA |
|  | Upper 95% CI | 3.01E+07 | NA | NA | NA | 2.86E+07 | NA |
