# Supplementary figures and images for: Repurposing the antimalarial pyronaridine tetraphosphate to protect against Ebola virus infection
Source: PLoS Negl Trop Dis. 2019 Nov 21;13(11):e0007890. doi: 10.1371/journal.pntd.0007890 (PMC6894882; doi:10.1371/journal.pntd.0007890)

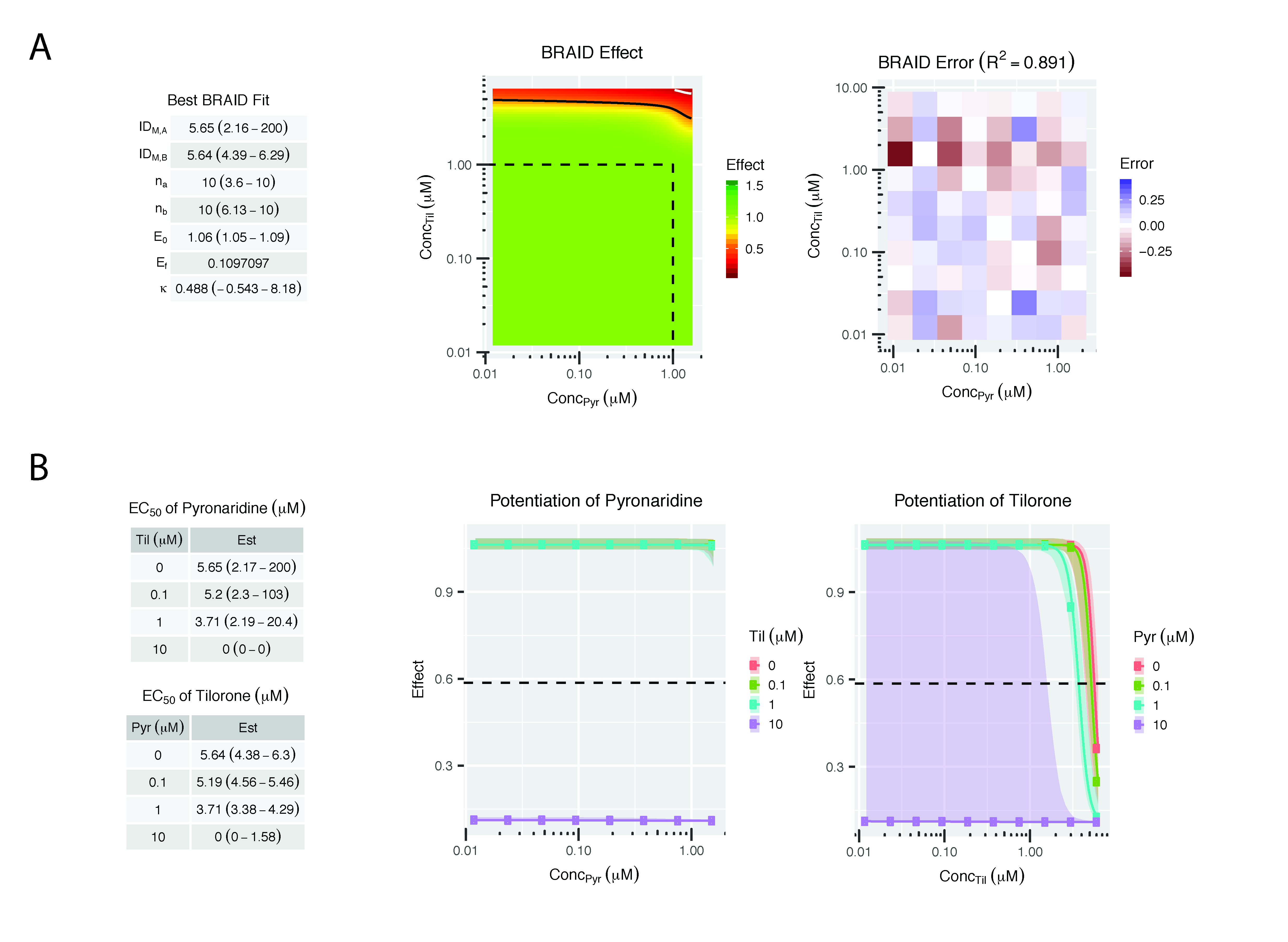

Supplement: S1 Fig — The variable κ represents a quantitative synergy value. κ < 0 implies antagonism, κ = 0 implies additivity, and κ > 0 implies synergy. The other variables are E0, the estimated effect when neither drug is present; Et, the maximal effects of either drug alone; IDM,A and IDM,B concentrations representing the EC50 of either drug alone; and na and nb, are the Hill equation parameters representing the sigmoidicity of both drugs’ dose response curves. A) The BRAID effect is the plot of the best BRAID fit. The BRAID error is the difference between the smoothed data and the best BRAID fit. The higher the R2 the better the fit the BRAID fit is to the raw, smoothed data. B) The potentiation of drugs is the interpolated effect curves for the drugs in combinations using the best BRAID fit equation. Data represents a checkboard assay with pyronaridine and tilorone at various combined concentrations (Fixed pyronaridine/tilorone concentrations of 0.012, 0.024, 0.049, 0.098, 0.195, 0.391, 0.781, 1.562, 3.125, 6.25, 12.5, or 25 μM) in HeLa cells. A calculated κ = 0.488 (- 0.543–8.18) suggested that these compounds are synergistic to each other for the inhibition of EBOV in HeLa cells. The BRAID analysis shows potentiation of the EC50 of tilorone by pyronaridine, but due to toxicity the potentiation of pyronaridine by tilorone could not be accurately analyzed. (TIF) [file pntd.0007890.s006.tif]

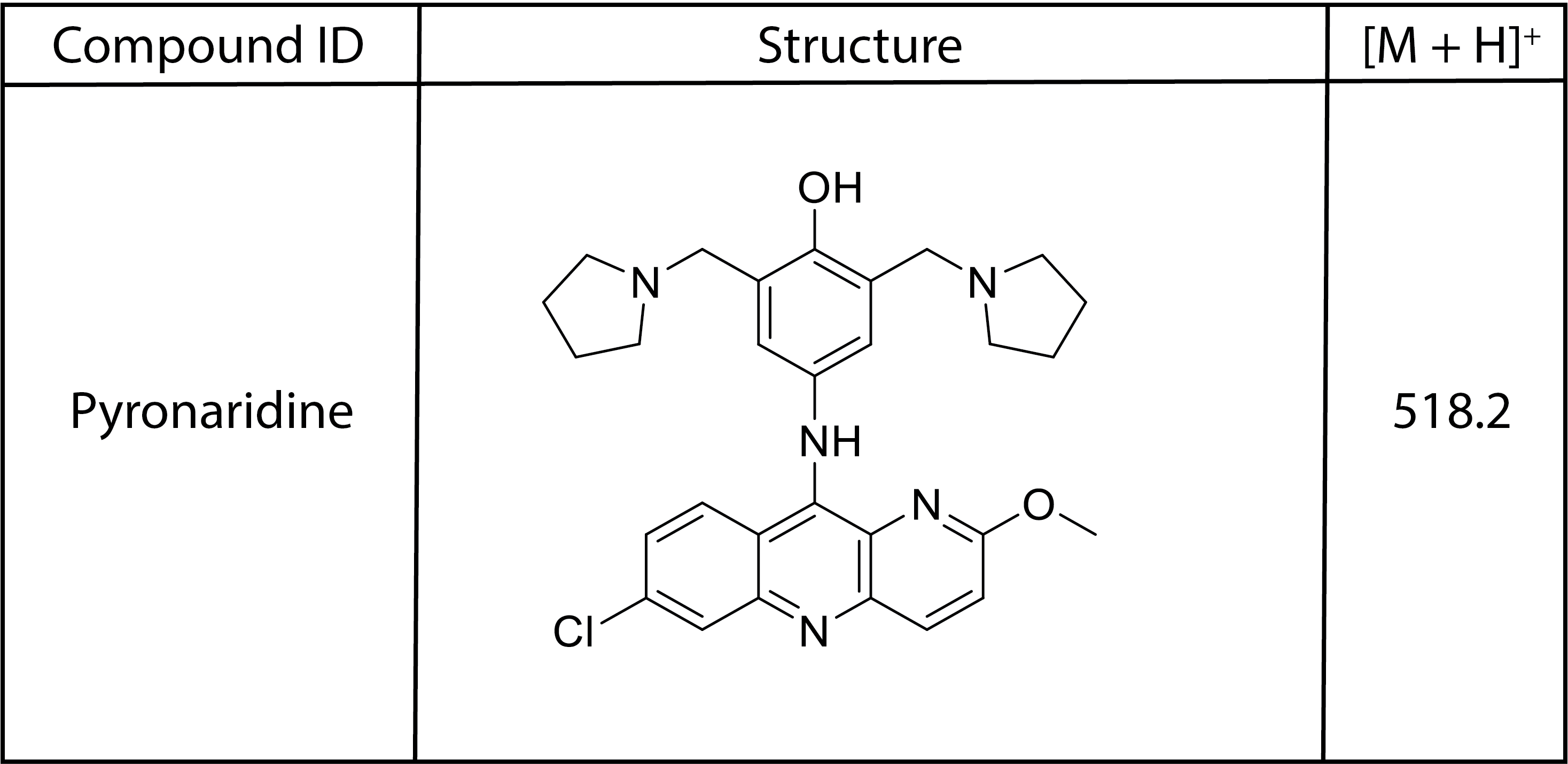

Supplement: S2 Fig — (TIF) [file pntd.0007890.s007.tif]

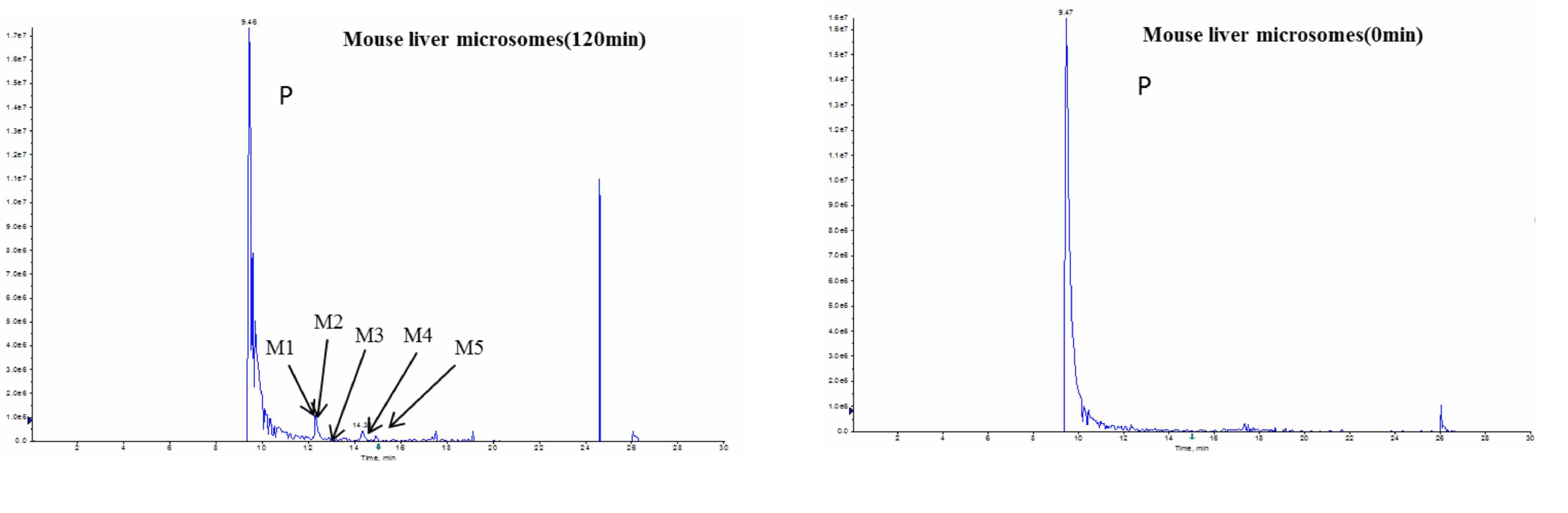

Supplement: S3 Fig — * For profiling only, each peak may have different ionization efficiency. *, in the incubation sample at 60 or 120 min, the abundance of potential metabolites (M1-M5) are normalized to that of parent drug (100%) based on the peak height. (TIF) [file pntd.0007890.s008.tif]

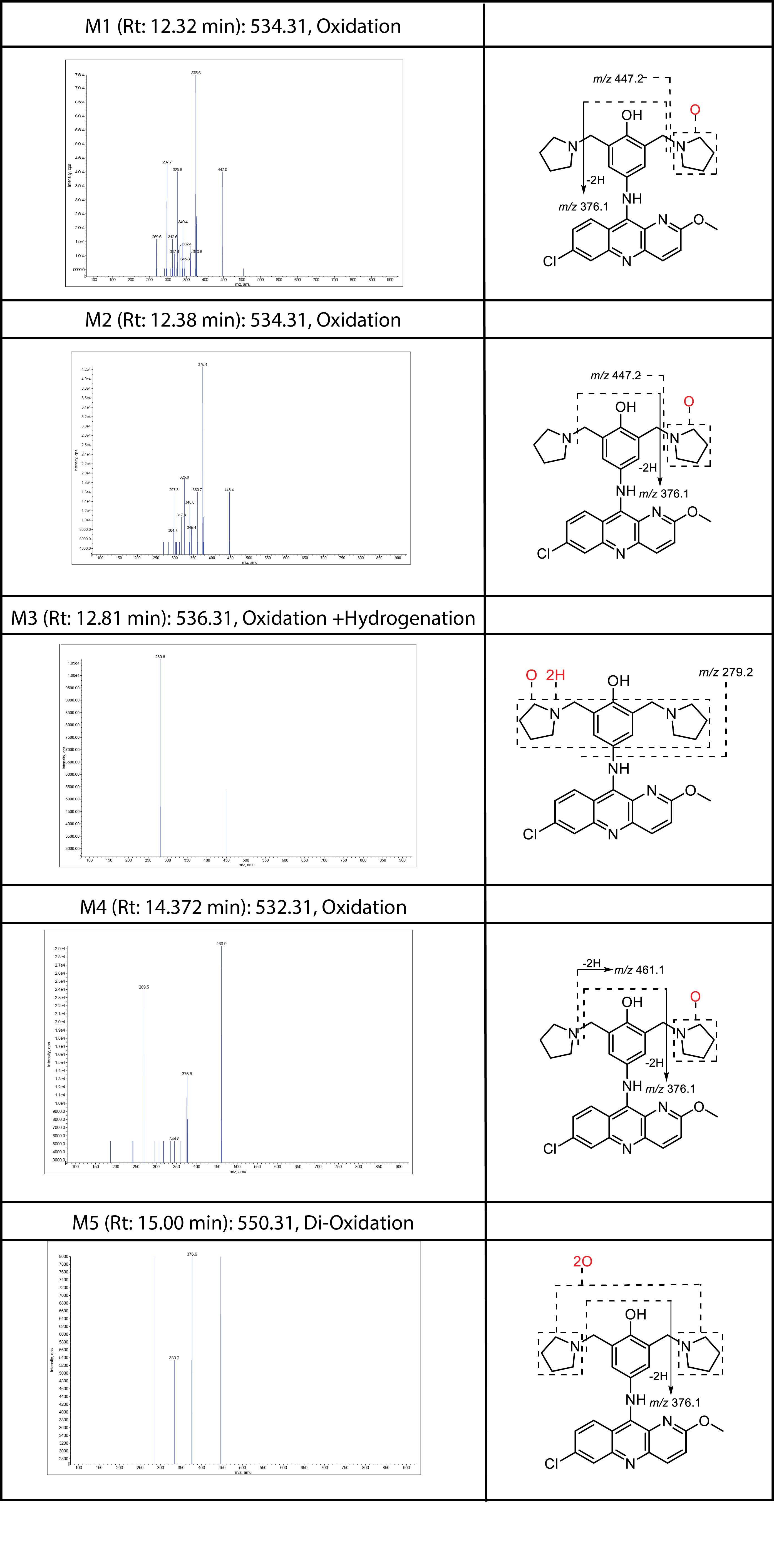

Supplement: S4 Fig — (TIF) [file pntd.0007890.s009.tif]

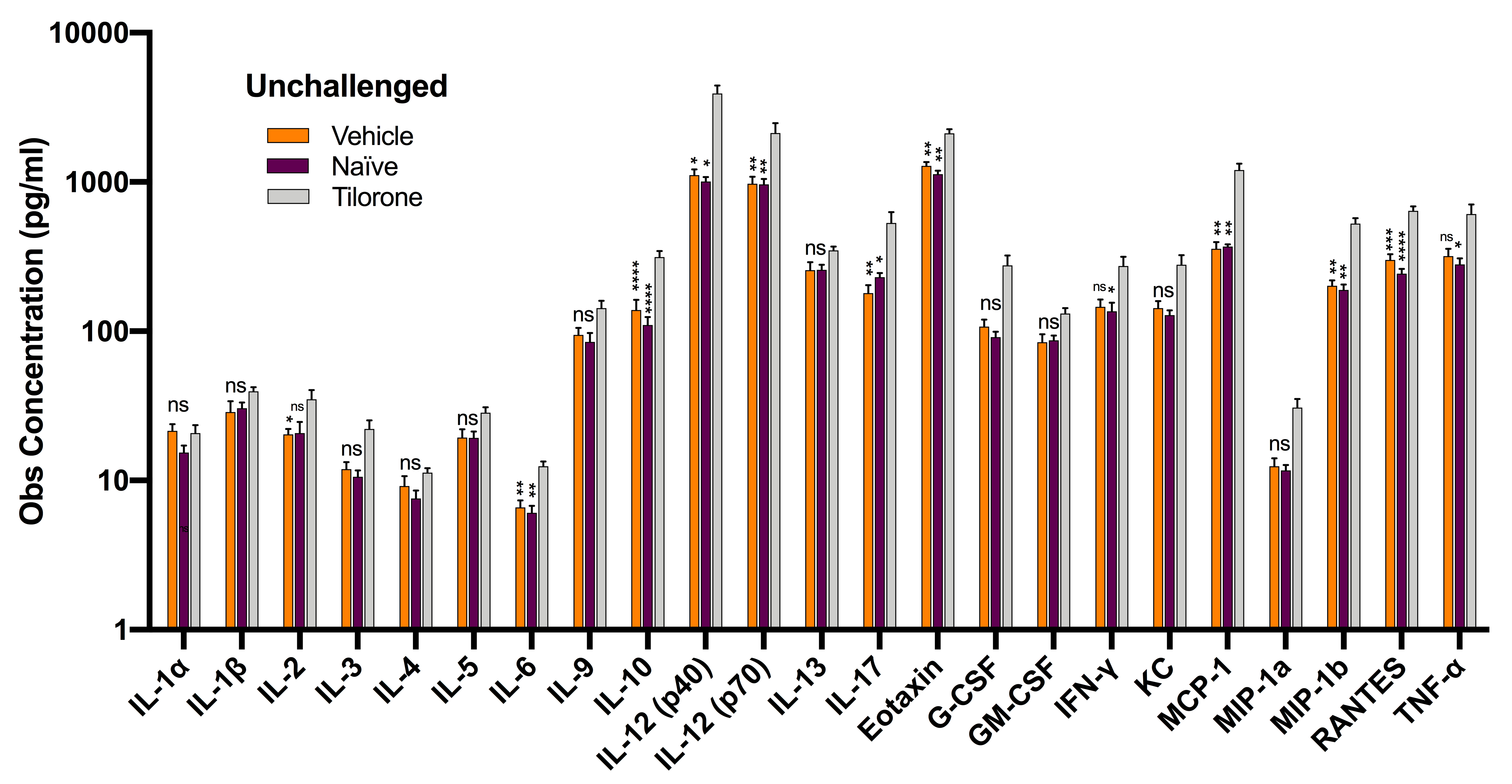

Supplement: S5 Fig — Each group was unchallenged (vehicle, naïve and tilorone) with each group comprised of 4 mice. Bars represent the mean and the error bars represent the SEM. Stars represent the significance of the difference from the unchallenged, tilorone-treated group (Tukey test). (0.0021< * ≤ 0.0332, 0.0002< ** ≤ 0.0021, 0.0002< *** ≤ 0.0001, **** < 0.0001). (TIFF) [file pntd.0007890.s010.tiff]

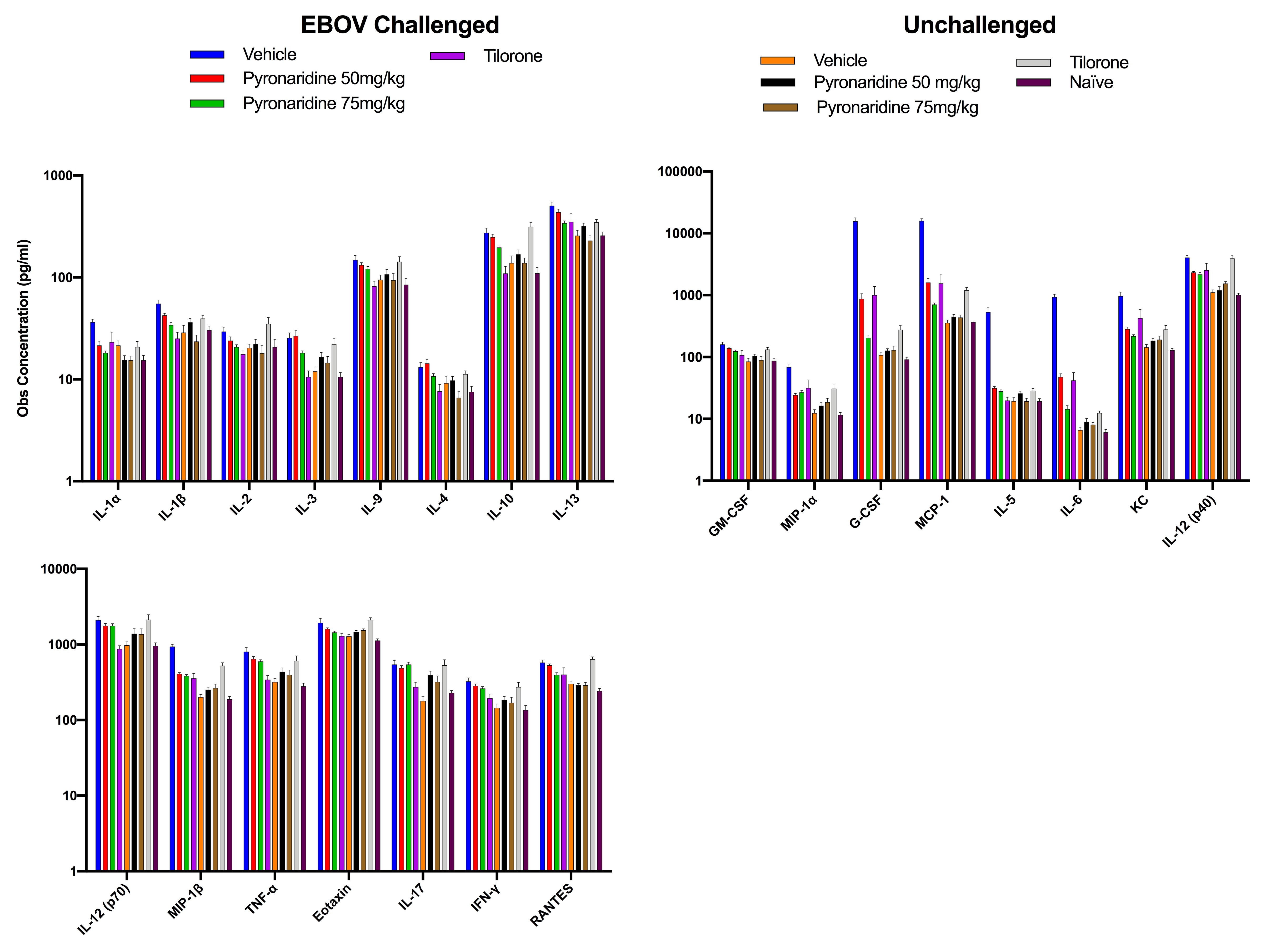

Supplement: S6 Fig — The observed concentration of each cytokine/chemokine was from either challenged (EBOV) or unchallenged (UN) mice, with the serum from each euthanized mouse run in duplicate. Each challenged vehicle (EBOV) and tilorone (EBOV) group comprised of 6 mice, while the pyronaridine 50 and 75 mg/kg (EBOV) groups had 12 mice each. The unchallenged groups each had 4 mice each. Error bars represent the SEM. (TIFF) [file pntd.0007890.s011.tiff]
