# Supplementary material for: Repurposing the antimalarial pyronaridine tetraphosphate to protect against Ebola virus infection
Source: PLoS Negl Trop Dis. 2019 Nov 21;13(11):e0007890. doi: 10.1371/journal.pntd.0007890 (PMC6894882; doi:10.1371/journal.pntd.0007890)
Supplement: S1 Supplemental Text and References — (DOCX) [file pntd.0007890.s012.docx]

**S1 Supplemental Text and References.**

It has been well established that EBOV triggers a robust immune response *in vivo* (1-3), with increases in levels of eotaxin, granulocyte-macrophage colony stimulating factor (GM-CSF), granulocyte colony-stimulating factor (G-CSF), IL-1β, IL-6, IL-8, IL-10, IL-15, IL-16, IL-18, IFN-γ, IFN-beta, MCP-1 (also known as CCL2), MIP-1α (also known as CCL3), MIP-1β (also known as CCL4), RANTES, TGF-1β and TNF-α (1-11). This immune response has also been potentially implicated in its viral transmission. Study of the pathogenesis of EBOV has also suggested that monocytes, macrophages and dendritic cells are the initial targets of EBOV *in vivo* (8, 12). Following the infection of these cells they release proinflammatory cytokines and chemokines attracting additional macrophages to the region of infection, thus locally increasing the number of cells that are prone to infection (13). While the activation of the immune system by EBOV may contribute to pathogenicity, it has also been shown that the EBOV proteins VP35 and VP24 suppress type I IFN expression and IFN signaling, respectively, indicating that a controlled immune activation is key for efficient EBOV viral propagation. It has been suggested that VP35 inhibits the RIG-I pathway, suppressing IFN-α/ß production, and then VP24 ultimately suppresses the downstream nuclear import of activated STAT1 (11, 14), indicating that early activation of the innate immune system may still be able to suppress viral proliferation.

**Supplemental References**

1. Reynard S, Journeaux A, Gloaguen E, Schaeffer J, Varet H, Pietrosemoli N, Mateo M, Baillet N, Laouenan C, Raoul H, Mullaert J, Baize S. 2019. Immune parameters and outcomes during Ebola virus disease. JCI Insight 4.

2. Baize S, Leroy EM, Georges AJ, Georges-Courbot MC, Capron M, Bedjabaga I, Lansoud-Soukate J, Mavoungou E. 2002. Inflammatory responses in Ebola virus-infected patients. Clin Exp Immunol 128:163-8.

3. Leroy EM, Baize S, Volchkov VE, Fisher-Hoch SP, Georges-Courbot MC, Lansoud-Soukate J, Capron M, Debre P, McCormick JB, Georges AJ. 2000. Human asymptomatic Ebola infection and strong inflammatory response. Lancet 355:2210-5.

4. Mahmud-Al-Rafat A, Majumder A, Taufiqur Rahman KM, Mahedi Hasan AM, Didarul Islam KM, Taylor-Robinson AW, Billah MM. 2019. Decoding the enigma of antiviral crisis: Does one target molecule regulate all? Cytokine 115:13-23.

5. Panchal RG, Mourich DV, Bradfute S, Hauck LL, Warfield KL, Iversen PL, Bavari S. 2014. Induced IL-10 splice altering approach to antiviral drug discovery. Nucleic Acid Ther 24:179-85.

6. Hensley LE, Young HA, Jahrling PB, Geisbert TW. 2002. Proinflammatory response during Ebola virus infection of primate models: possible involvement of the tumor necrosis factor receptor superfamily. Immunol Lett 80:169-79.

7. Leroy EM, Baize S, Debre P, Lansoud-Soukate J, Mavoungou E. 2001. Early immune responses accompanying human asymptomatic Ebola infections. Clin Exp Immunol 124:453-60.

8. Geisbert TW, Hensley LE, Larsen T, Young HA, Reed DS, Geisbert JB, Scott DP, Kagan E, Jahrling PB, Davis KJ. 2003. Pathogenesis of Ebola hemorrhagic fever in cynomolgus macaques: evidence that dendritic cells are early and sustained targets of infection. Am J Pathol 163:2347-70.

9. Herbert AS, Davidson C, Kuehne AI, Bakken R, Braigen SZ, Gunn KE, Whelan SP, Brummelkamp TR, Twenhafel NA, Chandran K, Walkley SU, Dye JM. 2015. Niemann-pick C1 is essential for ebolavirus replication and pathogenesis in vivo. MBio 6:e00565-15.

10. Falasca L, Agrati C, Petrosillo N, Di Caro A, Capobianchi MR, Ippolito G, Piacentini M. 2015. Molecular mechanisms of Ebola virus pathogenesis: focus on cell death. Cell Death Differ 22:1250-9.

11. Messaoudi I, Basler CF. 2015. Immunological features underlying viral hemorrhagic fevers. Curr Opin Immunol 36:38-46.

12. Connolly BM, Steele KE, Davis KJ, Geisbert TW, Kell WM, Jaax NK, Jahrling PB. 1999. Pathogenesis of experimental Ebola virus infection in guinea pigs. J Infect Dis 179 Suppl 1:S203-17.

13. Zampieri CA, Sullivan NJ, Nabel GJ. 2007. Immunopathology of highly virulent pathogens: insights from Ebola virus. Nat Immunol 8:1159-64.

14. Basler CF, Amarasinghe GK. 2009. Evasion of interferon responses by Ebola and Marburg viruses. J Interferon Cytokine Res 29:511-20.
